# Maintaining Shell Disorder with Kinked or Branched Ligands Stabilizes Apolar Nanoparticles

**DOI:** 10.1021/acsnano.5c12697

**Published:** 2025-09-26

**Authors:** Tobias Valentin Knapp, Setare Dodange, Debora Monego, Camila Martinez Briones, Devid Hero, Bart-Jan Niebuur, Markus Gallei, Tobias Kraus, Asaph Widmer-Cooper

**Affiliations:** † INM—Leibniz Institute for New Materials, Campus D2 2, Saarbrücken 66123, Germany; ‡ ARC Centre of Excellence in Exciton Science, School of Chemistry, 4334 University of Sydney, Sydney 2006, New South Wales, Australia; § Polymer Chemistry, 9379 Saarland University, Campus C4 2, Saarbrücken 66123, Germany; ∥ Saarene, Saarland Center for Energy Materials and Sustainability, Saarbrücken 66123, Germany; ⊥ Colloid and Interface Chemistry, Saarland University, Campus D2 2, Saarbrücken 66123, Germany; # The University of Sydney Nano Institute, The University of Sydney, Sydney 2006, New South Wales, Australia

**Keywords:** nanoparticles, colloidal stability, ligand structure, ligand shell, agglomeration temperature, apolar, nonlinear

## Abstract

Understanding how nanoparticles form stable colloids is fundamental to their practical applications. Nonlinear ligands are known to increase the stability of nanoparticles in apolar solvents compared to shells of linear alkyl chains. Here, we reveal the molecular origin of this colloidal stability. We observe that even a single methyl side chain can suppress disorder–order transitions in the ligand shell, with double bonds or branches leading to drastic decreases in agglomeration temperature in such dispersions. Through a combination of temperature-dependent X-ray scattering and molecular dynamics simulations, we show that these simple structural modifications prevent ligand molecules from forming ordered bundles, maintaining shell disorder even at temperatures approaching solvent freezing. The absence of ligand order enhances colloidal stability by weakening attraction between the ligand shells via a combination of energetic and entropic factors. This mechanism extends dispersion stability by more than 100 K compared to linear ligands of equivalent length. Our findings provide a molecular-level explanation for the enhanced stability previously observed with branched and unsaturated ligands, offering an effective strategy for engineering nanoparticle dispersions that remain stable across broad temperature ranges.

## Graphical abstract

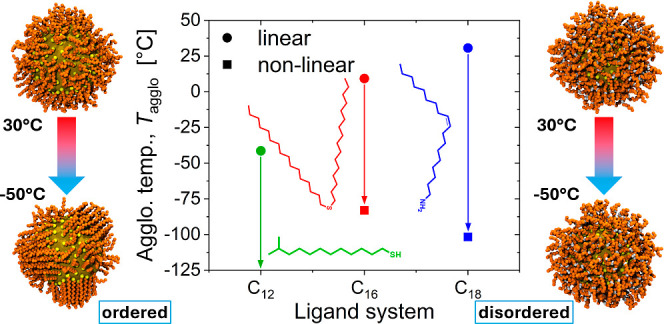

## Introduction

The development of stable colloidal nanoparticle dispersions has traditionally relied on various stabilization mechanisms, with electrostatic or steric repulsion through surface-bound ligands being key approaches.^1,2^ In apolar solvents, where electrostatic stabilization is negligible, steric repulsion has been commonly enhanced by increasing ligand length.^3^ However, this approach can have unexpected consequences. Upon cooling, or a change in solvent environment, linear ligands can undergo an ordering transition, where they align and pack together in ways that actually promote rather than prevent agglomeration.^4^ This temperature-dependent behavior has been observed across different systems, from CdSe nanoparticles with amine ligands^5^ to gold nanoparticles with alkanethiol shells,^6,7^ fundamentally challenging our understanding of colloidal stability. The ordered ligand state enhances van der Waals attraction between particles and reduces steric stabilization, leading to aggregation even under conditions where classical theories would predict stability.

Understanding these ligand-mediated interactions is crucial for applications in optoelectronics, catalysis, and biomedicine,^8–10^ where the unique size-dependent properties derived from quantum confinement must be preserved through precise surface functionalization.^11,12^ Beyond their stabilizing role, ligands guide nanoparticle growth, modify surface energy, and control interparticle interactions in dispersions.^13,14^ The complexity of these ligand-dependent phenomena underscores the need for a deeper understanding of shell organization principles to enable rational design of stable colloidal systems.

Various approaches have demonstrated success in enhancing colloidal stability. This includes modification of the solvent environment,^15,16^ the use of molecular additives,^17^ and changes to ligand composition and structure. For example, branched and mixed ligands have been shown to enhance stability at higher particle concentrations,^18,19^ with the argument made that this is related to their effect on the ligand shell structure and thermodynamics. While detailed observations of ligand shell packing have been reported,^20^ and correlations between shell crystallinity and solvent–ligand interactions have been proposed,^21^ the mechanistic relationship between shell organization and colloidal stability remains to be fully elucidated.

In particular, we lack a detailed understanding of how the ligand backbone structure, regardless of length, affects shell ordering and subsequent colloidal stability. While some correlations between ligand structure and stability have been established,^18^ the underlying thermodynamic mechanisms remain elusive, particularly regarding how structural features promote or inhibit shell ordering and how this affects the interaction between the particles.^22^ Features such as kinks, branches, and conformational constraints may influence these interactions through related mechanisms, highlighting the need for systematic investigation of structure–stability relationships.

In this study, we address this knowledge gap by examining how ligand backbone structure influences shell ordering and colloidal stability for apolar nanoparticles. By comparing linear, kinked, and branched ligands of similar lengths, we isolate the effects of backbone structure from those of ligand length. Through a combination of temperature-dependent small-angle X-ray scattering (SAXS) and molecular dynamics (MD) simulations, we establish clear relationships between ligand structure, shell ordering, and particle–particle interactions. Our findings reveal how ligand backbone structure can be used to maintain shell disorder and achieve robust colloidal stability across diverse environments.

## Results and Discussion

We experimentally compared the colloidal stability of gold nanoparticles (AuNP) with core diameters of 3.9–4.4 nm and 8.0–8.8 nm and ligand shells composed of three linear and three nonlinear ligands in *n*-hexane. All dispersions contained particles at concentrations of 2.5 mg/mL. Temperature-dependent small-angle X-ray scattering (SAXS) was used to quantify particle agglomeration upon cooling until either all particles had agglomerated or the solvent froze. Solvent freezing was detected using concurrent wide-angle X-ray scattering (WAXS). Pure *n*-hexane in SAXS capillaries froze between −105 °C and −110 °C due to supercooling.

The linear alkylthiols serve as reference systems for the branched and kinked ligands. Linear alkyl shells of 1-hexadecanethiol on AuNP with diameters between 4 and 10 nm have been studied previously. Such shells undergo a disorder–order transition that drives the agglomeration of “shell-dominated” particles that have cores with diameters below 8–9 nm.^6^ For example, 4 nm core AuNP with 1-hexadecanethiol shells agglomerates at 8 °C in hexane.^15^ We denote them as “AuNP@SC_*n*_” in the following, where *n* is the number of carbon atoms in the chain.

### Oleylamine as a “Kinked” Ligand

Oleylamine (OAm, (*Z*)-octadec-9-en-1-amine) is commonly used as ligand^23^ in the synthesis of spherical gold^24^ and silver^25^ nanoparticles and gold nanowires.^26^ We compared the stabilizing effect of this kinked ligand with 18 carbon atoms and a double bond at position C_9_ with its saturated analog, octadecylamine.

Figure 1a shows the temperature-dependent SAXS patterns of AuNPs with 3.9 nm diameter cores coated with octadecylamine (AuNP@NC_18_). Scattering at 50 °C was dominated by an intensity plateau at *q* < 0.05 Å^–1^ and decreased in intensities above, followed by an intensity maximum at *q* = 0.2 Å^–1^ to 0.3 Å^–1^ from the form factor of spherical particles.^27^ The plateau indicates the lack of particle–particle correlations in a fully dispersed sample.

**Figure 1:**
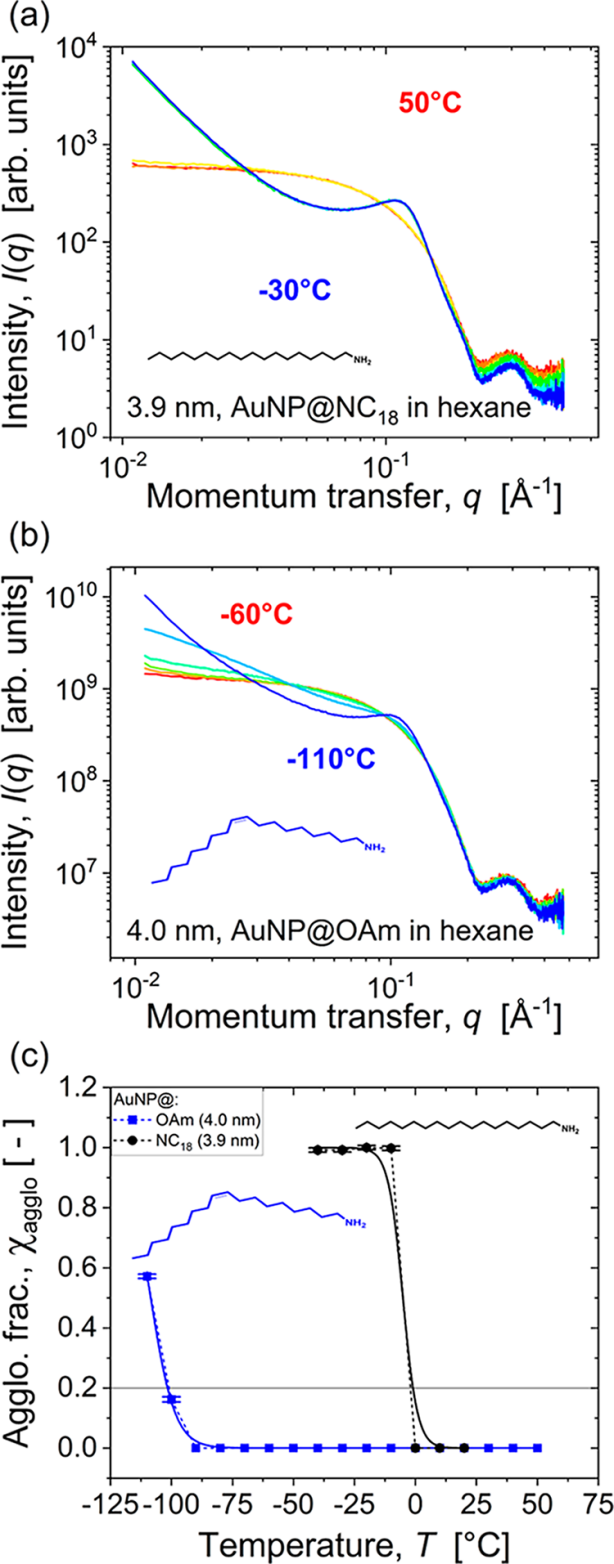
Temperature-dependent stability of AuNP with ≈4 nm diameter cores with octadecylamine and oleylamine shells as quantified by SAXS. Scattering patterns were recorded while cooling dispersions in *n*-hexane from 50 °C to −30 °C in steps of 10 °C for particles coated with (a) linear octadecylamine, and from 50 °C to −110 °C in steps of 10 °C for particles coated with (b) kinked oleylamine ligands with the same number of carbon atoms per ligand. (c) Fraction of AuNPs within agglomerates, χ_agglo_, in dependence on temperature as determined using model fits according to eq 10 in the Supporting Information.

Cooling the AuNP@NC_18_ dispersion to −30 °C led to the emergence of a peak at *q*_0_ ≈ 0.1 Å^–1^. This structure factor peak indicates spatial correlation of AuNPs that is caused by agglomeration. We used it to quantify the temperature-dependent stability of the dispersion following a previously published approach:^6,7^ A model-based analysis of the temperature-dependent scattering (see Supporting Information for details) yielded the fraction of AuNPs within agglomerates, χ_agglo_.

χ_agglo_ increased nonlinearly when cooling below *T* < 40 °C, following a sigmoidal trend (Figure 1c, black circles). We fitted it using eq 10 in the Supporting Information. Following literature,^6^ we defined the agglomeration temperature, *T*_agglo_, as the temperature at which the sigmoidal fit reached χ_agglo_ = 0.2 and found *T*_agglo_ = −0.9 °C.

Previous stabilization studies with this method focused on thiol ligands.^7^ Here, we find that the binding group has a moderate effect on colloidal stability: The agglomeration temperature of AuNP@NC_18_ was approximately 30 K below that of AuNP@SC_18_ (see detailed data below and in Figure S7 in the Supporting Information) and approximately 20 K below that of 6 nm CdSeNP with linear SC_18_.^7^ This moderate increase of stabilization through the more weakly bonded amines is likely an effect of reduced shell density and greater lability, as we discuss in more detail in the section below on ligand shell densities.

The stabilizing effect of unsaturated “kinked” ligands was considerably larger. Figure 1b shows the temperature-dependent X-ray scattering of OAm-coated 4.4 nm diameter gold cores (“AuNP@OAm” (Figure 1b)). The scattering at 40 °C was almost identical to that of AuNP@NC_18_, but it remained unchanged down to −90 °C, in stark contrast to the linear AuNP@NC_18_. The agglomeration temperature of AuNP@OAm was −101.6 °C. A single double bond thus increased the temperature-dependent stability of the dispersion drastically by more than 100 K.

### Branched Ligands

We now consider branched ligands of similar length. As a minimal case, we synthesized 11-methyldodecanethiol (cf. Methods) with a methyl side group at the second to last position of the main dodecyl chain. At the other extreme, we considered the “double-armed” dihexadecylsulfide with two hexadecyl chains connected to the sulfur atom.

Figure 2 shows the temperature-dependent agglomeration fraction of AuNPs with 4.4 nm diameter cores and ligand shells of 11-methyldodecanthiol (AuNP@*br*-SC_12_) and dihexadecylsulfide (AuNP@*dbr*-SC_16_). The same data for linear alkylthiols of the same lengths (AuNP@SC_12_ and AuNP@SC_16_) are shown as references.

**Figure 2:**
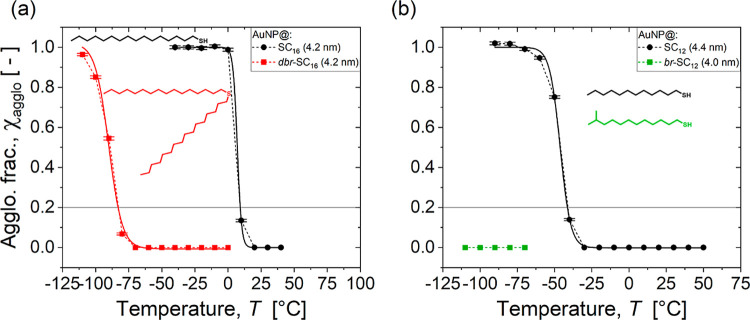
Temperature-dependent agglomerate fractions of AuNP with ligand shells composed of (b) linear SC_16_ and double-armed *dbr*-SC_16_ ligands with 16 carbon atoms in the chains (core diameter of both systems 4.2 nm), (b) linear SC_12_ (core diameter of 4.4 nm) and branched *br*-SC_12_(core diameter of 4.0 nm) with 12 carbon atoms in the main chains. Solid lines represent sigmoidal fits to eq 10 in the Supporting Information.

Branched ligands consistently reduced *T*_agglo_ by at least 70 °C. For example, χ_agglo_ remained at zero down to −70 °C for AuNP@*dbr*-SC_16_ and did not exceed 0.96 before the solvent froze (at temperatures below −110 °C), while AuNP@SC_16_ had fully agglomerated already at 0 °C. We found *T*_agglo_ of −83 and 9.2 °C for AuNP@*dbr*-SC_16_ and AuNP@SC_16_, respectively. Remarkably, the minimally branched AuNP@*br*-SC_12_ did not agglomerate at all before the solvent froze, while its linear counterpart AuNP@SC_12_ had an agglomeration temperature of *T*_agglo_ = −41.3 °C.

### Ligand Shell Densities

Differences in ligand structure lead to ligand shells with different densities. The ligand shell density is known to affect the colloidal stability of nanoparticles. Simulations predicted a reduction of the ordering temperature of octadecanethiol ligands on CdSe nanorods by 50 K when the ligand density was decreased by 25%.^28^ If the ligand density becomes too low, the repulsive interactions of the shell can no longer compensate for the attractive interactions of the cores, resulting in agglomeration.^29,30^ Yamashita et al. compared the surface coverages of linear and nonlinear organophosphonic acids on 4 nm ZrO_2_ and 5 nm TiO_2_ nanoparticles. Nonlinear 1-octyldecyl phosphonic acid and 1-butyltetradecyl phosphonic acid had only 65–70% of the surface densities of linear analogs. It was shown that the reduction in coverage in the case of linear ligands leads to a deterioration in colloidal stability. In contrast, a change in surface coverage is significantly better compensated by nonlinear ligands.

We performed thermogravimetric analysis (TGA) to determine the ligand shell densities and analyze whether they are responsible for the observed differences in colloidal stability. The results are summarized in Figure 3a and Table 1 (cf. Figure S14 in the Supporting Information). The ligand density of AuNP@OAm was 4.6 nm^–2^, similar to that of AuNP@NC_18_ (5.0 nm^–2^). The density of the corresponding alkylthiol SC_18_ was 30% higher (6.7 nm^–2^). The branched ligand AuNP@*br*-SC_12_ had a density 5.1 nm^–2^. This value corresponds to 91% of the linear reference AuNP@SC_12_ (5.6 nm^–2^). Given the two-armed structure of dihexadecylsulfide, it is more useful to compare the density of its alkyl chains on the surface; this density was 5.2 nm^–2^, 83% of AuNP@SC_16_ (6.3 nm^–2^). The difference in shell density can be explained by the molecular structure, but also by the binding energy. The binding energy of thiols is generally higher (by a factor of 1.5 to 2) than that of sulfides.^31,32^ Unfortunately, there are hardly any binding energies in the literature that compare the adsorption of alkyl thiols and alkyl sulfides with the same chain length. However, the difference is significantly smaller than that between a thiol and an amine.

**Figure 3:**
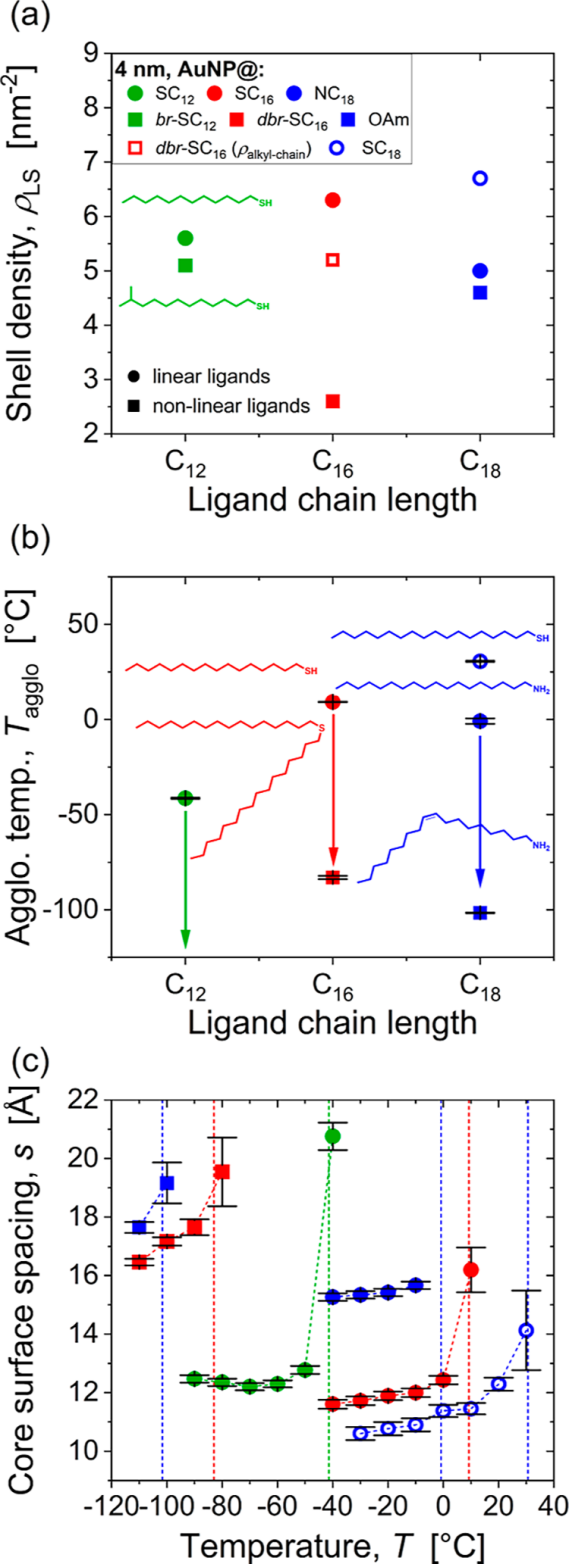
Structure and stability of AuNPs with core diameters of approximately 4 nm and different ligand shells. (a) Shell densities ρ_LS_ of linear and nonlinear ligands. (b) Agglomeration temperatures *T*_agglo_, where 20% of the particles were part of agglomerates. (c) Core-surface spacings *s* of particles in agglomerates. Spacings were calculated as *s* = 2 × (*r*_HS_ – *r*_core_) with *r*_HS_ from SAXS (cf. Supporting Information).

**Table 1:** Ligand Shell Densities ρ_LS_ of the Nonlinear Ligand Systems and the Linear Reference Systems

core diameter	4 nm	8 nm
ligand	ρ_LS_ (nm^–2^)	ρ_LS_ (nm^–2^)
OAm	4.6	3.8
NC_18_	5.0	3.6
SC_18_	6.7	6.9
SC_12_^br^	5.1	4.9
SC_12_	5.6	6.5
S(C_16_)_2_	2.6	2.0
SC_16_	6.3	7.5

The alkylthiol SC_18_ agglomerated at *T*_agglo_ = 30.6 °C, 30 K above the corresponding amine NC_18_. This difference is likely due to the higher ligand density of the thiol (6.7 nm^–2^ vs 5.0 nm^–2^ for the amine) that results from the lower binding energy of the amine.^33^ Reduced densities change the energy/entropy balance of the ligand shell, thus reducing the transition temperature where the ligands form ordered bundles and increasing colloidal stability.^6,7,28,34^

We investigated the shell structure by determining temperature-dependent, average, nearest-neighbor surface-to-surface distances *s* between particles from SAXS measurements. The structure factor of AuNPs within agglomerates was determined using a model-based fit. The interparticle distance was extracted and the average particle diameter subtracted to obtain *s* (see the SAXS section in the Supporting Information for details of this analysis). We note that this approach differs from that used in previous work,^6,7^ in which *s* was estimated using a simplified analysis and only at a temperature close to *T*_agglo_. The structural model used here is more established and believed to be more accurate; cf. the SAXS section in the Supporting Information for a detailed comparison between both methods. The absolute difference in the spacing values determined using the two analysis methods was 0.6 to 1 nm, and the new method consistently yielded smaller spacings. The temperature-dependent evaluation of *s* that we present here also provides a more nuanced picture of the shell structure during AuNP agglomeration.

Figure 3c shows that the linear ligands consistently resulted in smaller spacing than the respective (or any) nonlinear ligand shell. The determined values of *s* of all ligand systems were smaller even than the lengths of the respective linear ligands when assuming a stretched all-trans conformation and considering C–C bond length and C–C–C bond angle (17.1 Å for SC_12_, 22.8 Å for SC_16_ and 25.4 Å for NC_18_).^35,36^ It is known that linear ligands can form bundles on small NPs that can interlock and reduce *s*.^7,34^ Apparently, this mechanism is not active in nonlinear ligand shells, even in cases where their density is lower.

The increased spacing may contribute to the increased stability of such particles, but a difference in density of less than 10% cannot explain why the *br*-SC_12_ does not agglomerate before the solvent freezes.^28^ The high colloidal stability must therefore be the result of another shell property of the nonlinear ligands. At the same time, it is unclear why the spacings between kinked and branched ligand shells are much larger than for linear ones. In the following, we use Molecular Dynamics (MD) simulations to better understand the structural and thermodynamic origins of the enhanced colloidal stability and larger spacing observed for kinked and branched ligands.

### Temperature Dependence of Ligand Shell Structure

To separate the effect of ligand structure from shell density, we used a constant ligand density of 4.5 nm^–2^ in most of our simulations. This is similar to our experimental measurements for both kinked (4.6 nm^–2^) and branched (5.1 nm^–2^) ligands on 4 nm cores. While Yang et al. reported lower ligand densities (2 nm^–2^ to 3 nm^–2^) for branched thiols on CdSe nanocrystals,^18^ our chosen density allows a more direct comparison between simulation and experimental results for the systems studied here. To examine density-dependent effects, some additional simulations at lower surface coverage (3 nm^–2^) were performed for branched ligands.

We first examined the temperature-dependent behavior of various ligands on a 3.8 nm Au nanoparticle by quantifying their average dihedral angles in hexane (Figure 4). To separate the effect of ligand structure from ligand length, we limited the length of the longest ligand chain to 11 or 16 carbon atoms, but otherwise considered all structural variations present in the experimental ligands, including a kink in the middle of the chain, as well as branches at the binding group and near the end of the chain (structures at left). We found that the linear ligands (undecane and hexadecanethiol) exhibited a nonlinear transition to higher dihedral angles at reduced temperatures, consistent with the formation of ordered ligand bundles as described in previous studies of linear alkanethiol-coated nanoparticles.^6^ Ligands in such ordered shells are on average more extended with a larger proportion of trans dihedral angles and undergo fewer conformational changes than mobile disordered ligands, which results in a higher average dihedral angle in their tails (diagram at bottom right). In contrast, both kinked (*cis*-8-hexadecenethiol) and branched (6-pentylundecanethiol, 10-methylundecanethiol, and dihexadecylsulfide) ligands maintained lower dihedral angles throughout the temperature range, suggesting persistent disorder even at low temperatures. These differences in the behavior of shells composed of linear and nonlinear ligands can be clearly seen by comparing snapshots at high and low temperatures (top right of Figure 4), which show that the structure of the nonlinear shells hardly changes. This insensitivity of the shell structure to cooling aligns with our experimental observations where OAm-coated particles remained stable down to −101.6 °C and branched ligand systems showed no agglomeration before solvent freezing.

**Figure 4:**
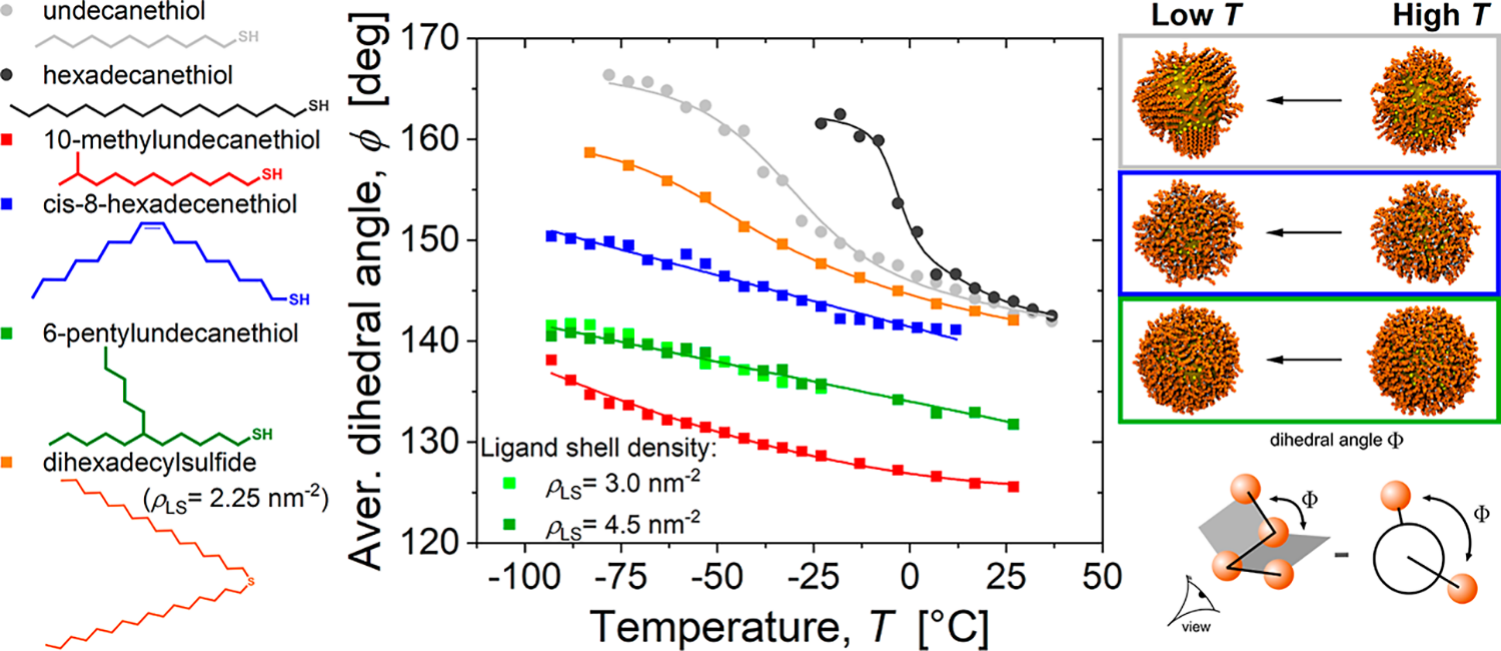
Average dihedral angle between CH_*x*_ groups for various ligands on 3.8 nm Au particles in hexane. This reveals an ordering transition upon cooling for linear ligands and the absence of one for nonlinear ligands. For comparison, simulation snapshots for some of the ligands are shown at temperatures that are high (23 °C for undecanethiol and 6-pentylundecanthiol, 7 °C for *cis*-8-hexadecenethiol) and low (−53 °C for all three ligands) with respect to the ligand ordering transition for undecanethiol. To clearly illustrate the trends in the values, curves for linear ligands were fit to a two-step sigmoid function, while curves for other ligands were fit to linear and polynomial functions.

Importantly, this disordered state proved to be a robust feature of nonlinear ligands, persisting across variations in molecular features, including different ligand lengths, branch points, and branch lengths (Figure S15b, Supporting Information). To isolate this intrinsic structural effect from solvent–ligand interactions, we performed additional simulations in the absence of solvent (Figure S15a, Supporting Information). Even at these conditions and at temperatures as low as −90 °C, almost all branched and kinked ligands remained disordered, in stark contrast with their linear counterparts. This persistence of disorder even when no ligand-solvent interactions are present indicates that there is simply no stable ordered state for most nonlinear ligands on small nanoparticles. Only dihexadecylsulfide was able to partially order in solution, forming small bundles composed of a single chain from each ligand (Figure S21), while 10-methylundecanethiol was the only additional ligand to order in vacuum (Figure S16).

### Particle Stabilization by Disordered Shells

Calculation of the potential of mean force (PMF) between pairs of nanoparticles in hexane (see Methods) showed that this prevention of ligand ordering has profound effects on interparticle interactions. Particles coated in linear hexadecanethiol ligands are already strongly attractive at −23 °C (Figure 5a) due to strong attraction between the ordered ligand bundles (red inverted triangles). Interdigitation of ordered bundles also allows the cores to get close together, consistent with the small core-surface spacings observed experimentally. In sharp contrast, particles coated in both kinked and branched ligands are still strongly repulsive at −53 °C (Figure 5b,c), with the disordered ligand shells preventing the cores from moving as close together. Even at −93 °C, the interaction between kinked ligand shells remains repulsive (Figure 5d), consistent with our experimental findings for OAm-coated particles. The main reason for the repulsive total interaction between nonlinear ligand shells (green circles) is that the ligand–ligand component (red inverted triangles) remains only weakly attractive, even at low temperature. And this, in turn, is due to the inability of the nonlinear ligands to access a densely packed ordered state.

**Figure 5:**
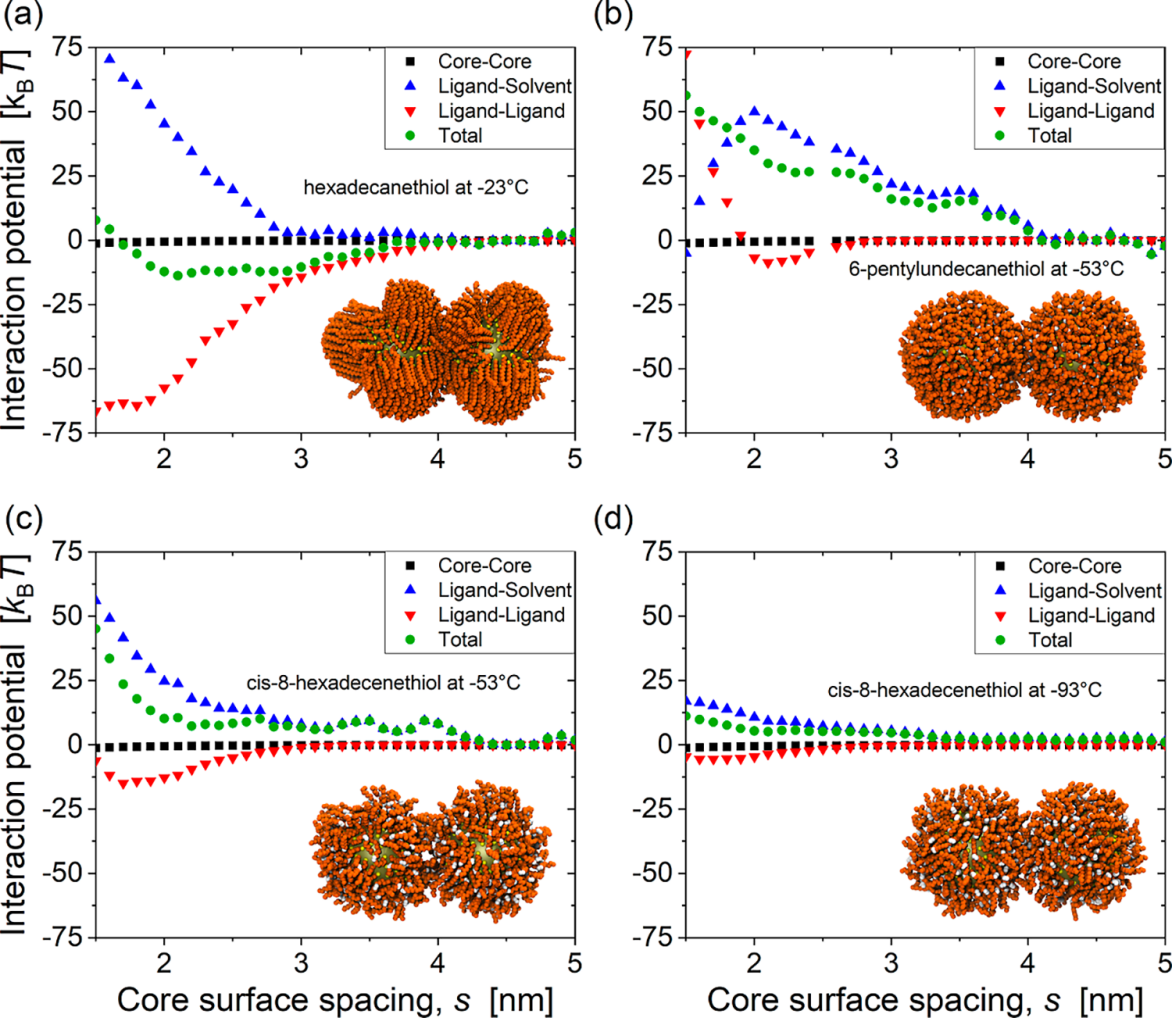
Potentials of mean force in hexane between 3.8 nm Au NPs coated with (a) hexadecanethiol at −23 °C, (b) 6-pentylundecanethiol at −53 °C, and *cis*-8-hexadecenethiol at (c) −53 °C, and (d) −93 °C. Results are shown separately for the total potential (green circles) and its ligand–solvent (blue triangles), ligand–ligand (red inverted triangles) and core–core (black squares) components. The inserts show simulation snapshots at a core surface spacing of 1.9 nm, with the solvent hidden for clarity.

We note that this conclusion differs from the argument made in a recent review,^21^ that the colloidal stability is due to stronger ligand–solvent interactions when ligands pack less efficiently. Calculation of the ligand–solvent interaction energies as a function of temperature for isolated particles (see Figure S17, Supporting Information), shows that there is indeed a decrease when the linear ligands order in hexane, but the absolute values of the ligand–solvent interaction energies are larger in some cases for linear ligands, even when those particles are not colloidally stable. We also note that in hexadecane, the ligand–solvent interaction energy continues to increase in magnitude upon ordering of hexadecanethiol ligands, which has the opposite effect of destabilizing the particles.^15^ So the magnitude of the ligand–solvent interaction does not appear to be the most important distinction between the colloidal stability of linear and nonlinear ligands.

The similar repulsive behavior observed in this work for both kinked and branched ligands indicates that preventing ligand ordering enhances colloidal stability regardless of the specific nonlinear ligand structure. These molecular-level findings help explain our experimental observations of remarkable stability of OAm-coated particles and the lack of agglomeration in branched ligand systems before solvent freezing. In the absence of agglomeration driven by ligand ordering, the particles remain colloidally stable until the remaining sources of attraction between them, i.e., the core–core vdW attraction and the ligand–solvent interfacial energy, become sufficiently large relative to the entropy of mixing to drive the particles together. The start of this change can be seen by comparing Figure 5c,d where the repulsive contribution due to ligand–solvent forces becomes weaker upon cooling.

### Conformational Entropy and Dynamics of the Ligand Shell

We further analyzed how ligand structure affects the thermodynamics and dynamics of the ligand shell, as several papers have argued that branched ligands enhance colloidal stability due to entropic effects. For example, Yang et al. proposed that branched ligands enhance stability by maximizing the intramolecular entropy of the ligand shell while reducing the thermodynamic stability of the crystalline state;^18^ while Elimelech et al. argued that conformational entropy differences between linear and branched thiol ligands contribute to the stabilizing effect of branched ligands on gold nanoparticles.^22^

For the analysis, we considered isolated particles in solution and calculated the vdW interaction between the ligands, and their conformational entropies, as a function of temperature. These results are shown in Figure 6, with conformational entropies quantified using the CENCALC software package^37^ (see the Methods section for details). The ligand–ligand interaction energies (shown in panel a) increase only slightly in magnitude upon cooling for most nonlinear ligands, whereas there are dramatic increases for the linear ligands as they order to form tightly packed bundles. The dihexadecylsulfide ligands exhibit intermediate behavior, as they partially order to form small bundles consisting of a single chain from each ligand (Figure S21). In contrast, the conformational entropies (shown in panel b) exhibit more subtle differences. While the branched ligands have a higher conformational entropy than their linear counterparts, the kinked C16 ligands have an entropy similar to that of linear C16 ligands in their disordered state. This shows that the main distinction between linear and nonlinear ligands lies not in the absolute values of the conformational entropies but rather in their temperature dependence: Linear ligands undergo a step-like entropy decrease coinciding with their ordering transition, while most branched and kinked ligands exhibit more gradual linear decreases upon cooling.

**Figure 6:**
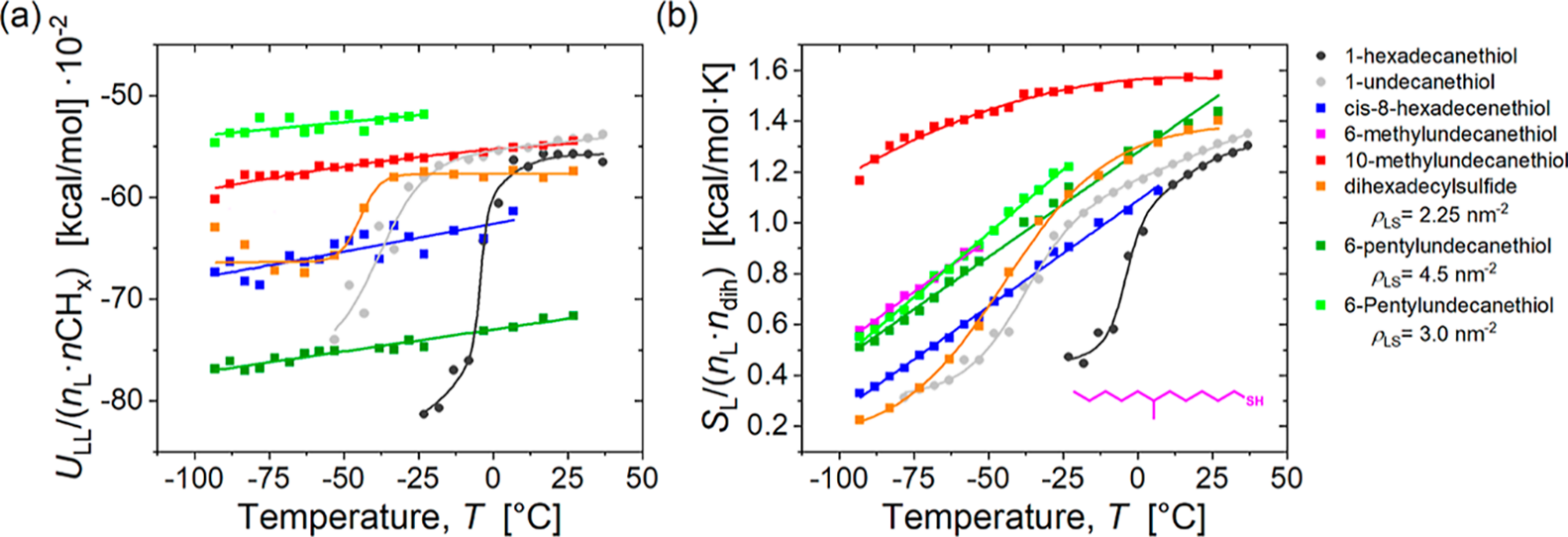
(a) Ligand–ligand interaction energy, and (b) conformational entropy of ligands on isolated 3.8 nm Au NPs in hexane. The energy values have been normalized by the number of carbon atoms in all ligands *n*_L_·*n*CH_*x*_ while the entropy values have been normalized by both the number of ligands *n*_L_ and the number of dihedral angles in each ligand *n*_dih_. To clearly distinguish the trends, we fitted the curves for linear ligands to a two-step sigmoid function, whereas the curves for other ligands were fitted to linear or polynomial functions.

Over a sufficiently large temperature range, though, most ligands lose the same amount of conformational entropy relative to the number of dihedral angles. Strikingly, the conformational entropy of the kinked C16 ligand shell at −93 °C is lower than that of the linear C16 ligand shell at −23 °C, yet the interaction between the former particles is repulsive (Figure 5d) while the interaction between the latter particles is attractive (Figure 5a). This shows that shell disorder can maintain colloidal stability (due to weak vdW attraction between poorly packed ligands) even when the conformational entropy of the ligands becomes very small.

Somewhat surprisingly, our results indicate that the molecular structure of the ligands, rather than their surface density, is the dominant factor in determining conformational entropy. For example, 10-methylundecanethiol exhibited notably higher conformational entropy than other ligands at all temperatures. In contrast, 6-pentylundecanethiol showed intermediate entropy values that only increased slightly when the surface coverage was reduced from 4.5 to 3 ligands/nm^2^. Reducing the size of the side chain at the 6-position, from a pentyl to a methyl group, had a similarly small effect on the conformational entropy (see Figure 6b), whereas moving that methyl group from the 6- to the 10-position, i.e., from the middle to the end of the chain, had a much larger effect. The reason for this behavior is that the ligand with a branch at the end of the chain retains more conformational flexibility at low temperature compared to the ligand with a branch near the middle of the chain, likely due to radial packing effects. This can be seen when comparing probability distributions for the dihedral angles near −85 °C, discretized into trans and gauche states (see Figure S20). The branch at the end of the chain results in the most uniform distribution and thus the highest conformational entropy, as the conformational entropy is related to the product of the probabilities of the dihedral states along the ligand chains. Geometrically, it would make sense that radial packing effects are responsible, as the high surface curvature of the approximately spherical 4 nm cores will provide more space for ligand motion when the branch is near the end of the chain. Comparison of our results with other recent work,^38^ however, suggests that the optimal branch position may vary with particle size and shape, or ligand length, although more work is needed to fully understand the effects of ligand structure on colloidal stability.

To further compare the dynamic nature of these ligands, we calculated the mean square displacement of the ligand atoms at several temperatures (Figure S18). As the ligands are not free to diffuse, this eventually reaches a plateau value characteristic of the conformational freedom of each ligand shell (Figure 7). These results show that all ligands become less mobile upon cooling, with a slower approach to the plateau, but that differences in ligand structure result in vastly different changes in the plateau value. For linear hexadecanethiol ligands, there is a large decrease in conformational freedom when they bundle together. In contrast, the conformational freedom of 10-methylundecanethiol ligands hardly changes upon cooling from 7 °C to −93 °C, consistent with the very weak temperature dependence of their conformational entropy.

**Figure 7:**
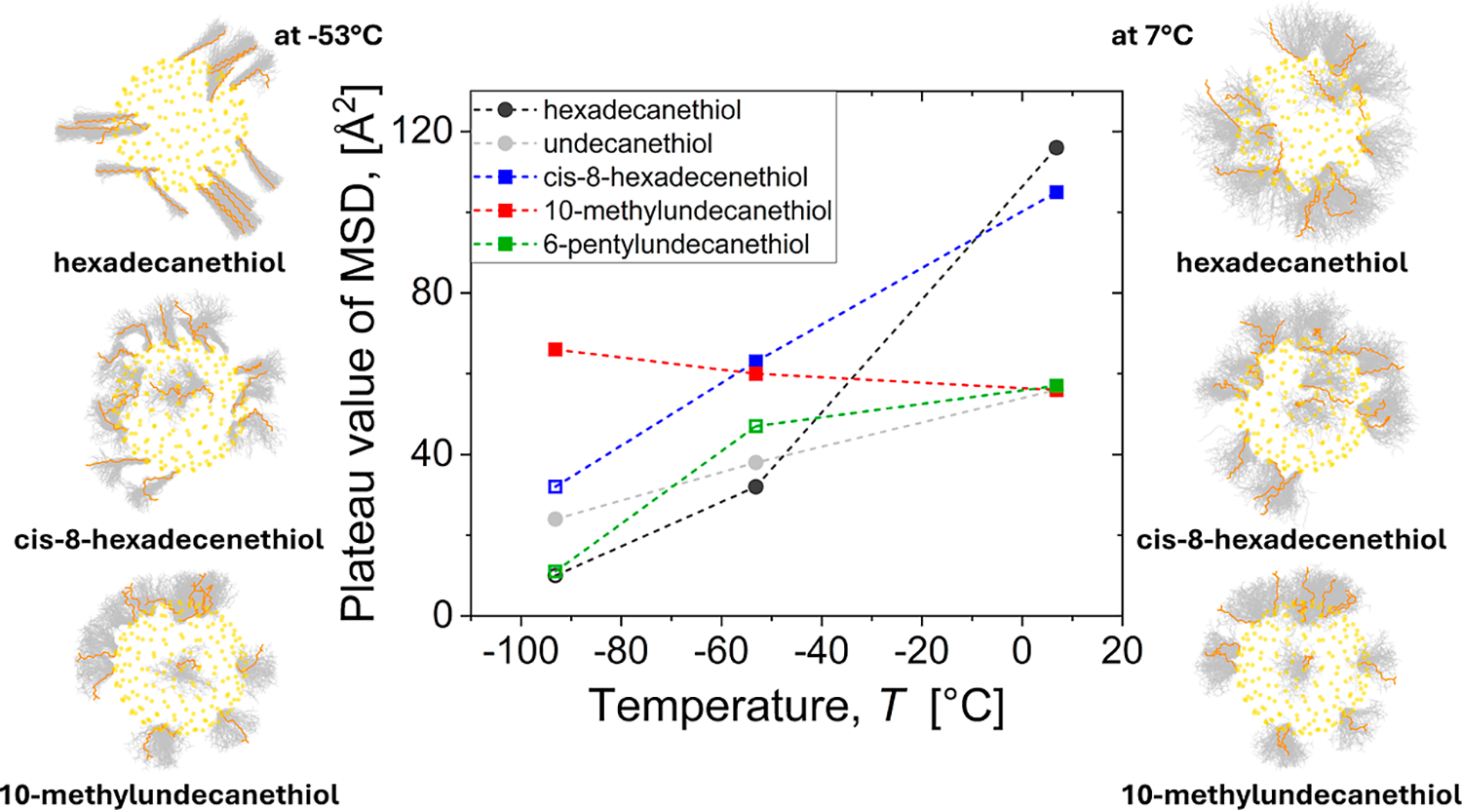
Conformational freedom of ligands on isolated 3.8 nm Au NPs in hexane as quantified by the plateau value in their mean square displacement versus time. Data points shown as open symbols are lower bounds corresponding to nonplateau values reached after 15 ns. The snapshots show trajectories of ligands with different sensitivities to cooling. Each snapshot is composed of 100 overlaid frames, captured at intervals of 0.025 ns.

Together, these results explain why even a single methyl group near the end of a linear tail can inhibit the ligand shell from ordering in hexane (as seen in our simulations) and enhance colloidal stability (as observed experimentally for particles covered with 11-methyldodecanethiol ligands). Interestingly, we find that the 10-methylundecane ligands do order in vacuum at around −100 °C (see Figure S16), i.e., they have the ability to pack into a mostly ordered state, but this only becomes thermodynamically favorable at low temperature in the worst possible solvent environment for our particles.

### Core Size Effects

The particles considered so far had gold cores with diameters below 5 nm. The van der Waals attraction between such cores is known to be weak enough that the stability is dominated by ligand ordering for linear octadecanethiol shells.^6,7^ The agglomeration of dodecanethiol-stabilized AuNPs with 9.0 nm cores, on the other hand, is known to be core-dominated and to occur well before the temperature-dependent transition in the ligand shell.^6,7^ Given our hypothesis that the increased stabilization by nonlinear ligands is connected to the inability of such ligands to pack together tightly, it is interesting to compare the shell-dominated cases with 4.4 nm diameter cores to larger cores, as we do in the following.

Figure 8 shows agglomeration temperatures and surface spacings of particles with diameters of approximately 8.5 nm. These cores were too large to be stabilized by linear or branched ligands with 12 carbon atoms below the solvent boiling temperature, indicating that nonlinear ligands do not substantially enhance colloidal stability in the core-dominated regime, i.e., when the vdW attraction between the cores is sufficiently strong (more than 2*k*_B_ *T* at 100 °C) to drive agglomeration on its own.^7^

**Figure 8:**
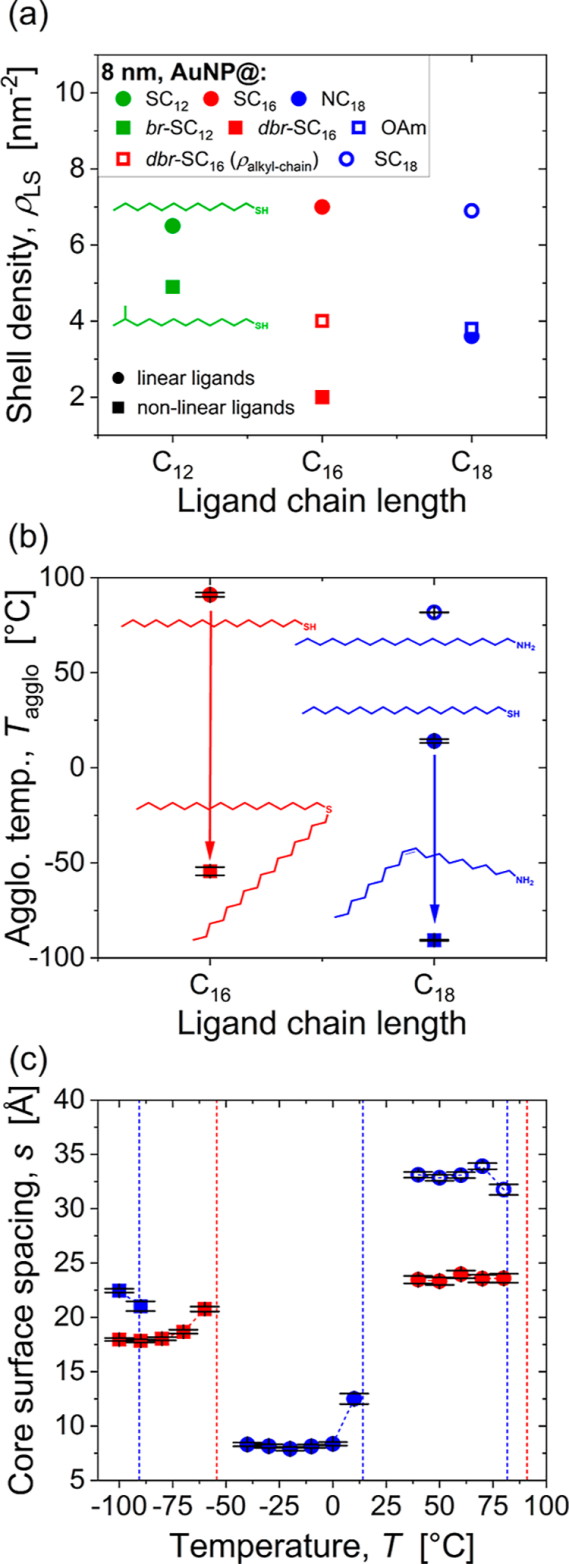
Structure and stability of AuNPs cores of approximately 8.5 nm diameter coated in different ligand shells. (a) Shell density ρ_LS_ of linear and nonlinear ligands (and chain density of the double-armed *dbr*-SC_16_). (b) Agglomeration temperatures *T*_agglo_, where 20% of the particles were part of agglomerates. (c) Core-surface spacings *s* of particles in agglomerates.

However, in the intermediate regime, where the ligands are long enough to limit vdW attraction between the cores to the order of *k*_B_ *T*,^7^ more complex behavior was observed as shown in Figure 8. Linear SC_16_ ligands yielded dispersions with *T*_agglo_ = 91 °C, 80 K above that of the respective 4.2 nm cores, whereas the longer linear NC_18_ had *T*_agglo_ = 14.0 °C, only 15 K above that for 3.9 nm cores. For the nonlinear ligands, similar but weaker effects of core size were observed: The agglomeration temperature of AuNP@*dbr*-SC_16_ increased by 29 K, from −83 °C for 4.2 nm to −54.4 °C for 8.5 nm cores, whereas the agglomeration temperature of particles with longer OAm ligands increased by only 10 K, from −101.7 °C for 4.2 nm to −90.6 °C for 8.5 nm cores.

The stability enhancement due to nonlinearity was also greater for these larger particles (up to 150 K) than for the smaller ones discussed earlier. This is surprising if one assumes that the destabilizing attraction of the cores is independent of the shell structure. This unexpected behavior appears to be related to changes in surface curvature and ligand coverage. The use of large cores increased the shell density for linear ligands (by up to 16% for AuNP coated with SC_12_) but consistently decreased it for nonlinear or amine ligands (by up to 30% for AuNP coated with *dbr*-SC_16_), such that the difference in shell density between linear and nonlinear ligands is greater for larger cores. This is likely explained by the “hairy ball effect” ^6,7^ that describes the geometrical arrangement of ligands on a highly curved surface. Whereas large gaps will form between linear ligands on highly curved, small cores, nonlinear ligands with a more conical overall shape can fill these gaps, allowing them to pack more densely on small cores than on large ones.

Increasing the core size, therefore, affects the colloidal stability in at least three ways, one largely independent of ligand structure (for a similar ligand length) and two strongly dependent on ligand structure. First, the increased vdW attraction between the larger cores increases *T*_agglo_ by 10 to 150 K, depending on the ligand length, with a larger increase for particles coated in shorter ligands. As shown in Figure 8c, the core spacings are now also more similar for linear and nonlinear ligands of similar length, probably due to the lack of gaps large enough to allow for interlocking of bundles formed by linear ligands. Second, the decreased surface curvature results in more substantial differences in ligand density between shells composed of linear and nonlinear ligands, with the nonlinear shells less dense than on smaller cores. This should enhance the relative stability of particles coated in nonlinear ligands, e.g., due to weaker vdW attraction between less dense ligand shells and greater solvent association with them, as long as the ligand shell is thick enough that the particles are not in the core-dominated regime. Third, the decrease in curvature increases the temperature at which shells composed of linear ligands order. For spherical cores, the expected increase from 4.2 to 8.5 nm is approximately 20 K at constant ligand density.^6^ Furthermore, larger gold cores are known to be faceted, with the (100) facets having higher ligand density,^39^ which should further increase the tendency of ligands to form ordered bundles.^28,40^ Lastly, it has been shown that extended particle contact, which is more likely to occur for the more attractive large cores, can induce ligand ordering at least 10 K above the ordering temperature for an isolated particle.^28^ The end result is that nonlinear ligands can enhance the stability of larger cores even more than small ones.

## Conclusions

A single double bond or methyl branch in the ligands of apolar nanoparticles drastically increases their temperature-dependent colloidal stability. We used X-ray scattering to show that the resulting difference in agglomeration temperature exceeds 100 K in many cases. The stability of many such colloids is not limited by agglomeration but by freezing of the solvent.

The superior stabilization by nonlinear ligands cannot be explained solely by differences in ligand shell density. Molecular kinks and branches that reduced ligand surface coverage by less than 10% increased stabilization drastically. Instead, their efficacy is due to large changes in the overall shell structure that are caused by small molecular differences.

Ligand shell disorder turns out to be the main prerequisite for stabilization. Molecular Dynamics (MD) simulations show that double bonds and branches stabilize a disordered ligand shell, which keeps the particles stable in solution even at low temperatures. Such nonlinear ligands inhibit the disorder–order transition that leads to the formation of attractive crystalline bundles in shells composed of linear ligands.

Nonlinear ligands also lose mobility upon cooling much more slowly than the corresponding linear ligands, which aids colloidal stability. In terms of conformational entropy, nonlinear ligands exhibit a diverse range of behavior, with some retaining high conformational entropy at low temperature and others not. However, even the less mobile, disordered state that they finally reach stabilizes nanoparticles better than the bundled state of linear ligands. Whereas linear ligands bundle and form areas of high ligand density that exclude solvent and strongly attract each other, nonlinear ligands usually do not form such areas, resulting in much weaker attraction between their ligand shells, even at temperatures where the conformational entropies are similar.

The stabilizing mechanism of nonlinear ligands cannot be understood at the single-molecule level alone. It is an emergent property of the interacting molecules in the ligand shell. The molecular dynamics in this shell deviates from the conventional bulk state as a result of the ligand-core bond. Stability is affected not only by the molecular ligand structure but also by the core size and geometry. For example, the presence of facets can result in differences in ligand density across the nanoparticle surface, while the relative orientation and size of facets influences the local order and dynamics of the ligand shell.^28^

While core-dominated agglomeration that is driven by the attraction between larger cores cannot be prevented by nonlinear ligand shells, their effectiveness extends into the transition region where the core alone cannot induce agglomeration. For example, oleylamine stabilized even cores with 8.5 nm diameter down to −90 °C.

Ligand shell disorder and mobility can be increased with other strategies, too. Mixed shells of linear ligands, for example, have been shown to dramatically enhance concentration-dependent stability while reducing the enthalpic gain of bundle formation.^21^ It will be interesting to assess whether they provide increased temperature-dependent stability as well, without the need for new molecular ligand structures.

## Methods

### Nanoparticle Synthesis

Gold nanoparticles stabilized by oleylamine and a core diameter of 4 nm were synthesized following a method described previously.^6^ A mixture of 9 mL of *n*-pentane (Carl Roth, 99%) and 9 mL oleylamine (Sigma Alrich, technical grade, 80–90%) was added to 100 mg of tetrachloroauric­(III) acid trihydrate HAuCl_4_·3H_2_O. The stirring time and the temperature used depend on the target particle size. Table 2 shows the parameter of the synthesis of the gold precursor for the different particle sizes. Then, a mixture of 1 mL *n*-pentane, 1 mL oleylamine and 40 mg *tert*-butylamine borane complex (Fluka, 97%) was added to the gold precursor solution. The mixture was stirred for 90 min. The dispersion was purified by precipitation with 50 mL of a mixture of ethanol and methanol (ratio 3:2) and centrifugation at 2000 rpm (689 rcf) for 5 min. The supernatant was removed, and the nanoparticles were redispersed in 2.5 mL toluene (gold concentration of 2.5 mg/mL).

**Table 2:** Conditions Used to Synthesize the Nanoparticles

temperature *T*, °C	time *t*, min	particle diameter	solvent
21	21	4 nm	*n*-pentane
21	2	8.3 nm (7 nm)	benzene

The larger particle with a diameter of 8.3 nm must be synthesized in two steps. First particles with a diameter of ∼7 nm were synthesized. These particles were dispersed in toluene and diluted to a gold concentration of 2.5 mg/mL (dispersion volume of 20 mL). The dispersion was heated to 60 °C. A solution of 60 mg of tetrachloroauric­(III) acid trihydrate HAuCl_4_·3H_2_O in 5 mL toluene and 1 mL was added to the warm dispersion. The mixture was stirred for 6 h at 60 °C. The dispersion was purified by precipitation with 50 mL of a mixture of ethanol and methanol (ratio 3:2) and centrifugation at 2000 rpm (689 rcf) for 5 min. The supernatant was removed, and the nanoparticles were redispersed in 2.5 mL toluene (gold concentration of 10 mg/mL).

### Synthesis of 11-Methyldodecanthiol

11-Methyldodecanthiol was synthesized by sequential synthesis of 11-methyldodecanol, 11-methyldodecyl bromide and 11-methyldodecanthiol following the scheme shown in Figure 9.

**Figure 9:**
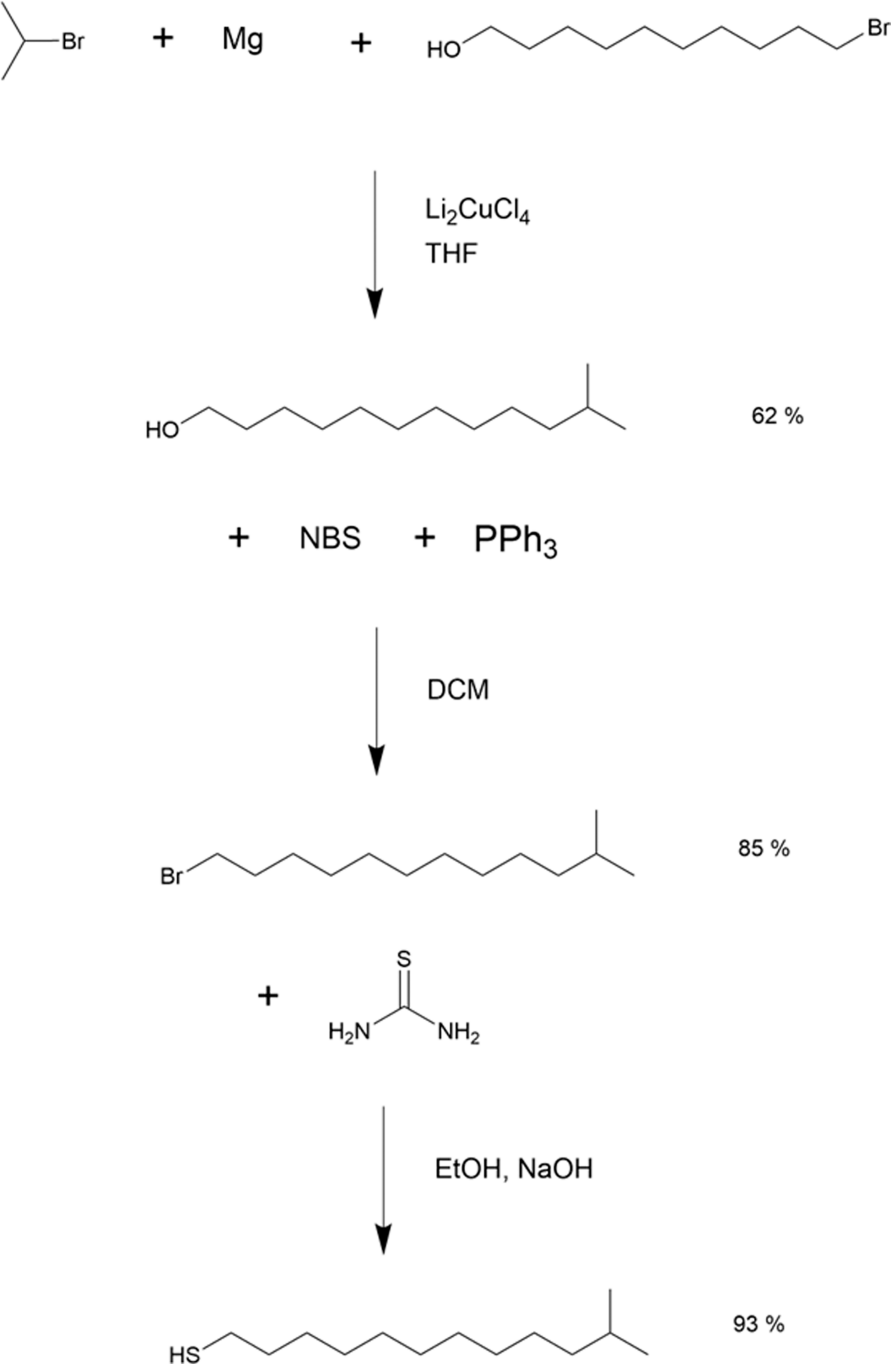
Synthesis of 11-methyldodecanthiol. In the first step 11-methyldodecanol was synthesized from 10-bromodecanol using *iso*-propyl and magnesium in a tertahyrofuran (THF) solution. In the second step 11-methyl bromide was synthesized using *N*-brom succinimide (NBS) and triphenylphosphine (PPh_3_) in a dichloromethane (DCM) solution. In the last step 11-methyldodecanethiol was synthesized using thiourea and sodium hydroxide (NaOH) in an ethanol (EtOH) solution.

#### 11-Methyldodecanol

To the *iso*-propyl magnesium bromide in THF solution (prepared from 4.25 g magnesium (173 mmol, 2.8 equiv) and 21.5 g isopropyl bromide (173 mmol, 2.8 equiv) in 100 mL THF), 21.6 mL Li_2_CuCl_4_ 0.1 M in THF (2.16 mmol, 3 M %) was added for 20 min at −25 °C and followed by stirring for 30 min. 14.65 g of 10-bromodecanol (61.77 mmol, 1 equiv) in 50 mL THF was added at −25 °C and the reaction mixture was then stirred for 3 h at the same temperature. After that time the mixture was added to 250 mL of 3 M HCl solution, stirred for 10 min, and extracted three times with 200 mL of diisopropyl ether. The combined organic layers were washed with 200 mL of brine and concentrated in vacuo. The residue was distilled under reduced pressure. 7.67 g of a colorless oil was obtained. Yield 62%, bp. 105 °C at 0.02 mbar.

#### 11-Methyldodecyl Bromide

To a solution of 7.50 g of 11-methyldodecanol (37.4 mmol, 1 equiv) and 11.87 g of triphenylphosphine (45.25 mmol, 1.21 equiv) in 50 mL of dichloromethane, 7.59 g of N bromosuccinimide (42.6 mmol, 1.14 equiv) was added in portions over 30 min maintaining the reaction flask at 4–8 °C. The reaction mixture was stirred for 30 min and followed by removal of the solvent by evaporation. The residue was diluted with hexane and the solid was removed by filtration and washed with hexane. The solution was concentrated and passed over a silica pad. After removing the solvent by evaporation, 8.37 g of the product was obtained as a colorless oil. Yield 85%.

#### 11-Methyldodecanthiol

9.25 g 11-methyldodecyl bromide (35.1 mmol, 1 equiv) was added to a solution of 3.02 g thiourea (39.6 mmol, 1.1 equiv) in 60 mL ethanol. The mixture was heated at reflux overnight. 1.59 g sodium hydroxide (39.6 mmol, 1.1 equiv) in 30 mL of water was added and the mixture was stirred at reflux for 2 h. The reaction mixture was cooled, poured into 100 mL of water and extracted three times with 250 mL dichloromethane. The organic layer was dried over MgSO_4_, filtered, and the solvent was removed in vacuo. 7.10 g of 11-methyldodecanthiol was obtained as a colorless liquid in a yield of 93.4%.

### Ligand Exchange

The ligand exchange was based on a previously published method.^41^ For the ligand exchange, the dispersion (2.5 mL, *c* = 10 mg/mL) was heated to 80 °C, followed by the addition of 1 mL hexadecanethiol (Sigma-Aldrich, GC, 95%) (0.4 mL per 1 mL of the concentrated dispersion, 1.3 M). The mixture was stirred for 15 min at 300 rpm. The dispersion was purified by precipitation with 3 times the sample volume (3 mL for 1 mL dispersion) of a mixture of ethanol and methanol (ratio 2:1) and centrifugation at 2000 rpm (689 rcf) for 5 min. The supernatant was removed, and the nanoparticles were redispersed in 2.5 mL toluene. The washing step was repeated once with a cooled mixture of ethanol and methanol (2:1). After the last washing step, the sample was redispersed in *n*-hexane and diluted to a gold concentration of 2.5 mg/mL.

Ligand exchange with 11-methyldodecanthiol and 1-dodecanethiol was performed in a similar way. A ligand concentration of 1.3 M was used. Due to the higher stability of the particles with the shorter ligands, higher rotation speeds were used during centrifugation (4000 rpm, 2755 rcf).

Ligand exchange with dihexadecylsulfide differed from classical exchange with thiols. Due to the limited solubility of this ligand, the concentration during the ligand exchange was reduced to 25 mg (dispersion volume of 2.5 mL) and the reaction time was extended to 20 min. For the purification, the nanoparticles must be precipitated without washing agents; an “optima XE-90” ultracentrifuge (Beckman Coulter, U.S.) was used for the precipitation of the nanoparticles. The dispersion was diluted to a volume of 4 mL and centrifuged at 60,000 rpm for 60 min. The supernatant was removed. The washing process was repeated once. The sample was dried and then redispersed in 2.5 mL of *n*-hexane.

Ligand exchange with octadecylamine was performed in a similar way. The ligand was added to reach a concentration of 1.3 M in a dispersion at 60 °C (0.89 g for 2.5 mL of the dispersion). The mixture was stirred for 15 min. The dispersion was purified by precipitation with 3 times the sample volume of ethanol and centrifugation at 2000 rpm (689 rcf) for 5 min. The washing step was repeated once. The completed ligand exchange and purification process was repeated twice. After the last addition of octadecylamine, the sample was washed three times. The particles were redispersed in *n*-hexane to a gold concentration of 2.5 mg/mL.

### Small- and Wide-Angle X-ray Scattering

SAXS measurements were performed on a Xeuss 2.0 instrument (Xenocs SA, Grenoble, France). The X-ray beam was produced by a copper Kα source (wavelength 1.54 Å) and focused on the sample with a spot size of 0.25 mm^2^. The measurements were performed with a sample–detector distance (SDD) of 1200 mm. The measurable momentum transfer *q* ranges from 0.01 Å^–1^ to 0.5 Å^–1^, with *q* being defined as *q* = 4π sin­(θ/2)/λ, where θ is the scattering angle.

The samples were measured in borosilicate capillaries with an inner diameter of 1.5 mm. For the temperature-dependent measurements, a “Linkam HFX350” temperature cell was used (Linkam Scientific Instruments Ltd., Redhill, United Kingdom). Before each temperature scan, the samples were equilibrated for 1 h in the capillaries, at a temperature well above *T*_agglo_ for the respective sample. During the temperature scans, the samples were cooled in steps of 10 °C to −90 °C or −130 °C. After each change in temperature, the samples were equilibrated for 5 min, followed by a SAXS measurement with an acquisition time of 15 min (3 times 5 min). The data were analyzed as described in the Supporting Information.

### Transmission Electron Microscopy

For the imaging of the gold nanoparticles, a “JEOL JEM2100 LaB_6_” electron microscope (JEOL ltd. Tokyo, Japan) with 200 kV acceleration voltage, 0.1 Å line resolution, and a Gatan Orius SC1000 camera (Gatan Inc. Pleasanton, CA, USA) in brightfield mode was used. AuNP samples were prepared by drop-casting of 10–20 μL of the particle dispersion in *n*-hexane (2.5 mg/mL) on a TEM copper grid. The analysis of the spacing between particles from TEM images is described in the Supporting Information.

### Thermogravimetric Analysis

For the TGA the samples were prepared by drop-casting of the nanoparticle dispersion into Al_2_O_3_ crucibles. All nanoparticle systems that were previously measured using SAXS were analyzed. The nanoparticle systems were transferred to *n*-hexane after the second washing step after the ligand exchange. The volume of pentane was smaller than the initial sample volume. Dispersions with concentrations of 20–30 mg/mL were produced.

The dispersions were transferred to the crucibles. The solvent *n*-pentane was evaporated at room temperature. After the evaporation at room temperature, the samples were dried in a vacuum oven (*p* = 10 mbar) overnight. For the TGA measurement a “STA 449F3” setup from NETZSCH was used. The samples were heated to 1000 °C over 90 min. During the heating, an argon atmosphere was used.

The densities of 8 nm SC_16_ and SC_18_ are at the upper possible limit given the binding group’s footprint (see Table 1) and suggests that a certain amount of free ligand was present.

### Molecular Dynamics Simulations

Our model, based on our previous work,^6^ consisted of a 3.8 nm Au nanoparticle core coated with various ligands, namely hexadecanethiol, undecanethiol, 6-pentylundecanethiol, 6-propylundecanethiol, 6-methylundecanethiol, 10-methylundecanethiol and *cis*-8-hexadecenethiol. The LigParGen web server^42^ was used to generate the initial topology for all ligands. Next, ligand molecules were positioned on the outside of a spherical shell with PACKMOL,^43^ resulting in our final nanoparticle systems. The NP core was modeled implicitly, with sulfur atoms of the ligands constrained to a shell 0.15 nm above the core surface using the RATTLE algorithm.^44^ Sulfur positions were optimized on this shell using a repulsive Coulombic potential (ϵ = 10 kcal mol^–1^, truncated at 24 Å) to ensure approximately equidistant binding sites. While 4 nm gold particles do have small facets, this spherical approximation has previously been used successfully to study ligand ordering on such particles, with good correspondence between the ligand ordering temperature and experimental agglomeration temperature for a wide range of ligand lengths and solvent types.^6,7,15,16^ The surface coverage was set to 4.5 ligands·nm^–2^, based on values measured experimentally by TGA. In view of lower values reported by previous work,^3^ we also investigated a lower coverage of 3 ligands·nm^–2^ for 6-pentylundecane ligands.

Ligands and hexane solvent were modeled using the united-atom TraPPE force-field,^45^ with each CH_*x*_ group treated as a single particle. Interactions between these groups were modeled using a 12–6 Lennard-Jones (LJ) potential, while CH_*x*_-core interactions used a 9–3 LJ potential (ϵ/*k*_B_ = 88 K, σ = 3.54 Å, truncated at 30 Å), as previously employed and described for similar particles.^4,7,46^ Bond stretching, bending, and torsion terms were included within each molecule.

Simulations were performed using the LAMMPS package,^47^ with periodic boundary conditions in all directions. Temperature and pressure were controlled using the Nosé-Hoover thermostat^48^ and Parrinello–Rahman barostat,^49^ respectively. While systems with linear ligands were simulated using a time step of 1 fs, branched and bent ligands required a reduced time step of 0.5 fs due to high densities within the ligand shell. To remove any high-energy configurations, we initially relaxed the solvated systems at constant energy for 10 000 ts. This was followed by a 1 ns constant volume (*NVT*) simulation at 300 K to stabilize the temperature. Subsequently, systems were equilibrated at high temperature (400 K) in the *NPT* ensemble to ensure disordered ligand states, followed by compression of the simulation box to match experimental solvent densities. The systems were then cooled to the desired temperature and equilibrated for at least 12 ns before production runs of 1 to 2 ns were performed. Molecular graphics were produced using Visual Molecular Dynamics (VMD).^50^

### Potential of Mean Force

To quantify interparticle interactions, we calculated the potential of mean force (PMF) for pairs of identical particles in explicit hexane using constrained MD simulations. This included 3.8 nm Au NPs coated with bent *cis*-8-hexadecenethiol or branched 6-pentylundecanethiol ligands at −53 °C. This temperature was chosen to investigate interparticle interactions where linear ligands typically exhibit ordered shells and strongly attractive interactions. We also repeated the procedure for NPs coated with the bent ligands at a lower temperature of −95 °C, close to the freezing point of hexane (*T*_*m*_ = −95 °C) and, therefore, to the lowest temperatures we could reach in our experiments.

The PMF was computed by gradually decreasing the interparticle separation from a noninteracting distance at a rate of 1 Å·ns^–1^. At each separation *r*, we allowed at least 10 ns for equilibration and sampling, with longer runs employed at lower temperatures due to reduced ligand lability. To improve configurational sampling, we reduced the nanoparticles’ moment of rotational inertia by 5% compared to the calculated value for solid gold spheres of their size. This modification allowed for faster rotational motion of the particles while maintaining physically reasonable behavior and energy conservation.

The interactions between the spherical Au cores were modeled using the Hamaker potential,^1^ with a Hamaker constant of 2 eV.^2^ This approach approximates the solvent and ligand environment as a continuous medium, with the interaction constant adjusted to account for the hydrocarbon environment.

The PMF was evaluated through numerical integration of the mean force between two particles in the direction of the line connecting them, calculated as

$$F_{mean}\left(r\right)=\frac{1}{2}\langle \left(\overset{⃗}{F_{2}\;}-\overset{⃗}{F_{1}\;}\right)\cdot {\mathrm{r⃗}\rangle }_{NVT}$$ 1

where $\vec{F_{1}\;}$ and $\vec{F_{2}\;}$ are the total forces acting on each NP, and *r⃗* is the unit vector connecting their centers.

### Conformational Entropy

We estimated the conformational entropy of ligand shells at various temperatures using the correlation corrected multibody local approximation (CC-MLA) method, as implemented in CENCALC.^37^ This approach captures the entropy contributions from different ligand conformations while accounting for correlations between dihedral angles within each molecule.

We represent the conformational space of each ligand by its dihedral angles, discretizing them into three locally stable states: *trans*, *gauche­(−)*, and *gauche­(+)*. This process transforms the continuous dihedral angle measurements into discrete random variables, allowing us to apply Shannon information entropy to quantify the conformational entropy of the ligands molecules

2 $$S_{conform}=-k_{B}\sum^{N}P\left(X\right)\ln\;P\left(X\right)$$

where *k*_B_ is the Boltzmann’s constant, *P*(*X*) is the probability mass function of the discretized conformational states, and *N* is the total number of possible configurations.

For our systems, direct application of this expression is impractical due to the large number of possible conformers (3^*M*^, where *M* is the number of dihedral angles) and would result in negatively biased entropies due to correlations between dihedral angles. To address these challenges, we employed the CC-MLA method,^37^ which provides an efficient way to estimate conformational entropy while accounting for genuine correlations between neighboring dihedral angles. The CC-MLA method improves upon traditional mutual information expansion (MIE) approaches by selecting an optimum distance-based cutoff that captures the maximum amount of genuine correlation while avoiding spurious correlations that can arise from limited sampling.

Converged estimates of the entropy were obtained by collecting data every 0.5 ps over 2.75 ns for branched and bent ligands and every 1 ps over 5.5 ns for linear ligands. To manage computational complexity due to the large number of molecules in our system, we treated each ligand independently and summed their individual entropies to approximate the entire nanoparticle’s ligand shell entropy. We note that, while this method captures intramolecular correlations, it neglects intermolecular ones, potentially underestimating entropy changes upon ligand ordering.

## Supplementary Material



## Notes

NMR spectra of 11-methyldodecanethiol synthesis, additional SAXS and WAXS results with discussion of fitting procedure, analysis of spacing in TEM images, TGA of ligand coverage, and additional simulation results (PDF)
