# Supplementary material for: Maintaining Shell Disorder with Kinked or Branched Ligands Stabilizes Apolar Nanoparticles
Source: ACS Nano. 2025 Sep 26;19(39):35127–40. doi: 10.1021/acsnano.5c12697 (PMC12509303; doi:10.1021/acsnano.5c12697)
Supplement: Supplementary file 1 [file nn5c12697_si_001.pdf]

Supporting Information for: Maintaining Shell Disorder with Kinked or Branched Ligands Stabilizes Apolar Nanoparticles

Tobias Valentin Knapp,[†,#] Setare Dodange,[‡,#] Debora Monego,[‡] Camila Martinez Briones,[†] Devid Hero,[¶] Bart-Jan Niebuur,[†] Markus Gallei,[¶,§] Tobias Kraus,[*,†,‖] and Asaph Widmer-Cooper[*,‡,⊥]

†*INM — Leibniz Institute for New Materials, Campus D2 2, 66123 Saarbrücken, Germany*

‡*ARC Centre of Excellence in Exciton Science, School of Chemistry, University of Sydney, Sydney, New South Wales 2006, Australia*

¶*Saarland University, Polymer Chemistry, Campus C4 2, 66123 Saarbrücken, Germany*

§*Saarene, Saarland Center for Energy Materials and Sustainability, 66123 Saarbrücken, Germany*

‖*Saarland University, Colloid and Interface Chemistry, Campus D2 2, 66123 Saarbrücken, Germany*

⊥*The University of Sydney Nano Institute, The University of Sydney, NSW 2006, Australia*

#*These authors contributed equally to this work*

E-mail: tobias.kraus@leibniz-inm.de; asaph.widmer-cooper@sydney.edu.au

Number of pages: 22

Number of figures: 21

Number of schemes: 0

Number of tables: 0

NMR data to synthesis of 11-methyldodecanthiol

The branched *br*-SC_{12} ligand 11-methyldodecanethiol was prepared in-house. The final product and the intermediates were characterized by NMR. The spectra are shown in Figure S1, S2 and S3.

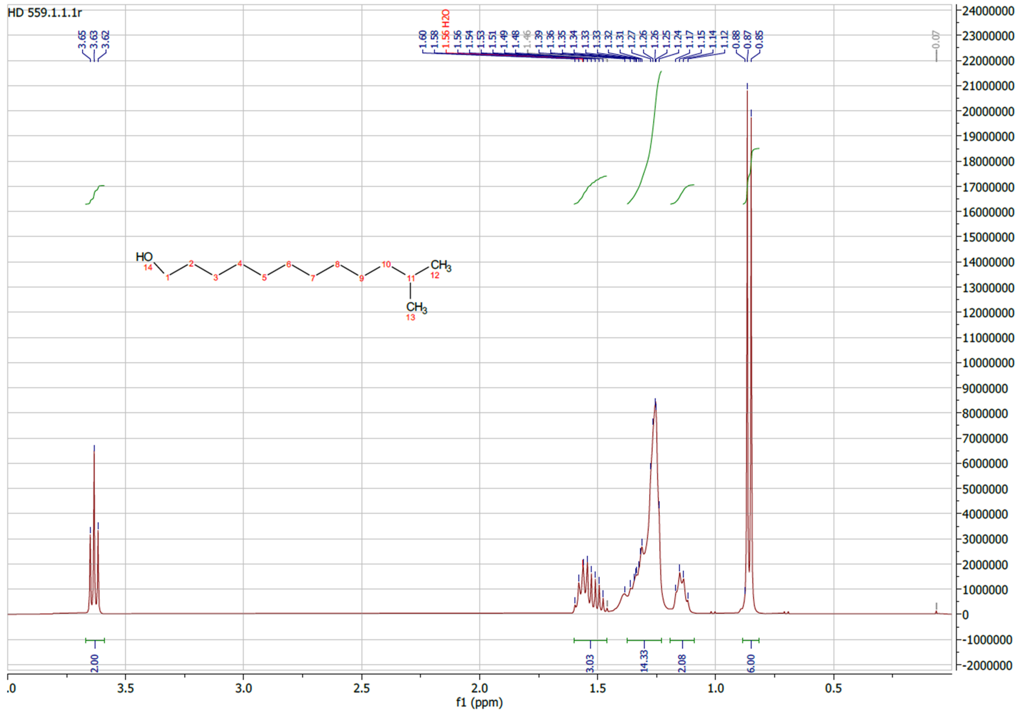

Figure S1: ^{1}H NMR of 11-methyldodecanol (400 MHz, CDCl3, δ in ppm): 3,63 (t, 2H, H1), 1.62-1.46 (m, 3H, H2+H11), 1.39-1.22 (m, 14H, H3-9), 1,15 (m, 2H, H10), 0.87 (d, 6H, H12+13).

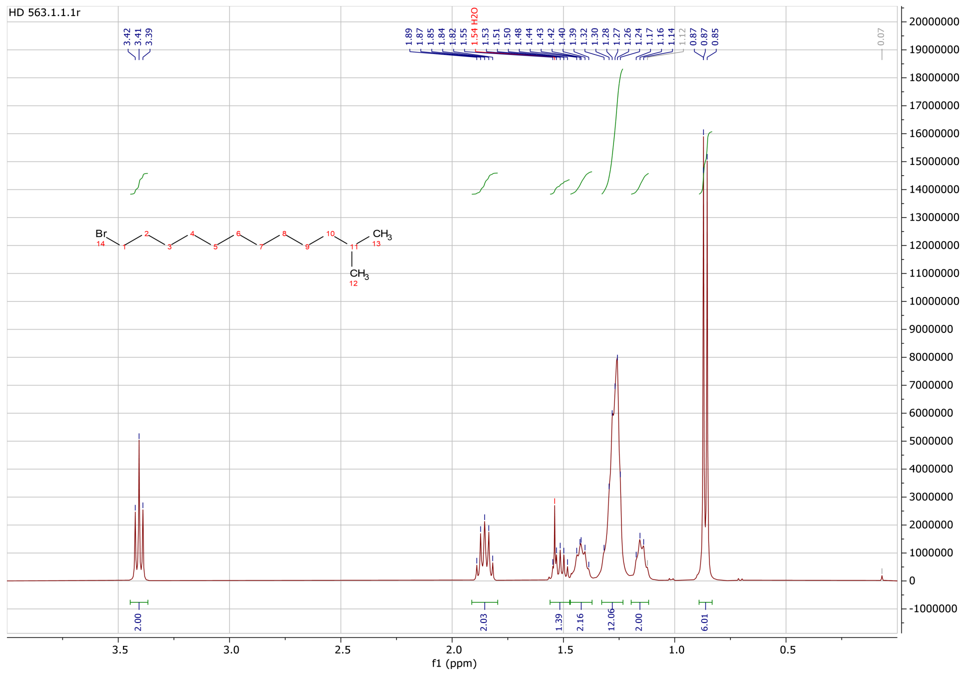

Figure S2: ^{1}H NMR of 11-methyldodecylbromide (400 MHz, CDCl3, δ in ppm): 2.41 (t, 2H, H1), 1.85 (q, 2H, H2), 1.51 (m, 1H, H11), 1.42 (m, 2H, H3), 1.31-1.22 (m, 12H, H4-9), 2.05 (m, 2H, H10), 0.87 (d, 6H, H12+13).

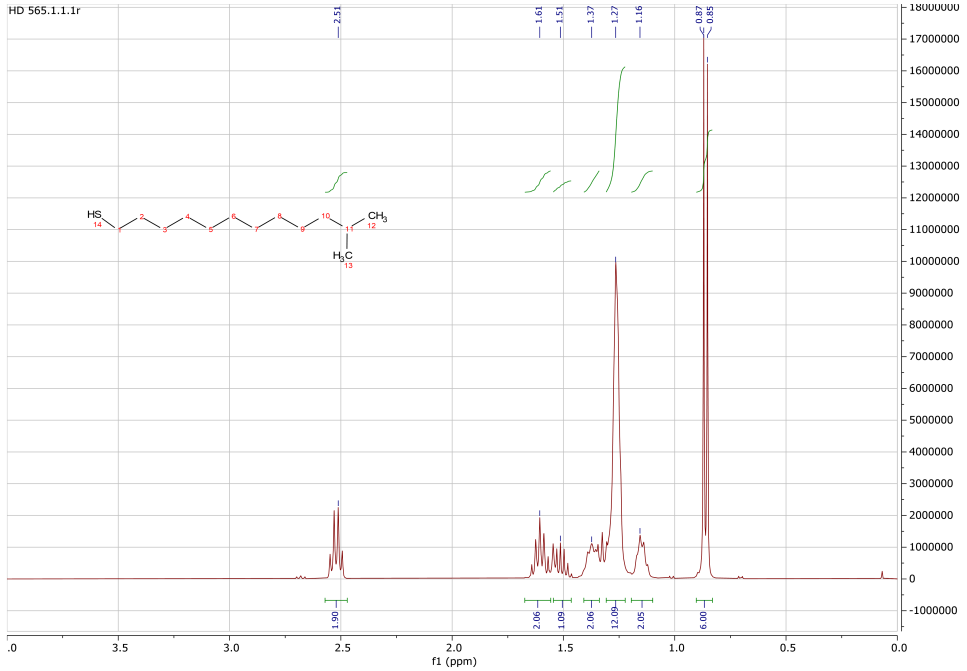

Figure S3: ^{1}H NMR of 11-methyldodecanthiol (400 MHz, CDCl3, δ in ppm): 2.51 (q, 2H, H1), 1.61 (m, 2H, H2), 1.51 (m, 1H, H11), 1.37 (m, 2H, H3), 1.31-1.22 (m, 12H, H4-9), 2.05 (m, 2H, H10), 0.87 (d, 6H, H12+13).

Small- and wide-angle X-ray scattering

The gold nanoparticles (AuNPs) used in this study were characterized using SAXS. Figure S4, S5 and Figure S6 show the scattered intensity, $I(q)$ of AuNPs in dependence on momentum transfer, q, in their dispersed state, i.e., the scattering pattern represents the form factor of AuNPs. In addition to the SAXS measurements, wide-angle X-ray scattering (WAXS) measurements were performed to detect freezing of the solvent, apparent from the presence of sharp structure factor peaks at q-values of 1-3 Å^{-1} (see Figure S5). In most samples, the solvent freezes at temperatures of around $-110\,°$C.

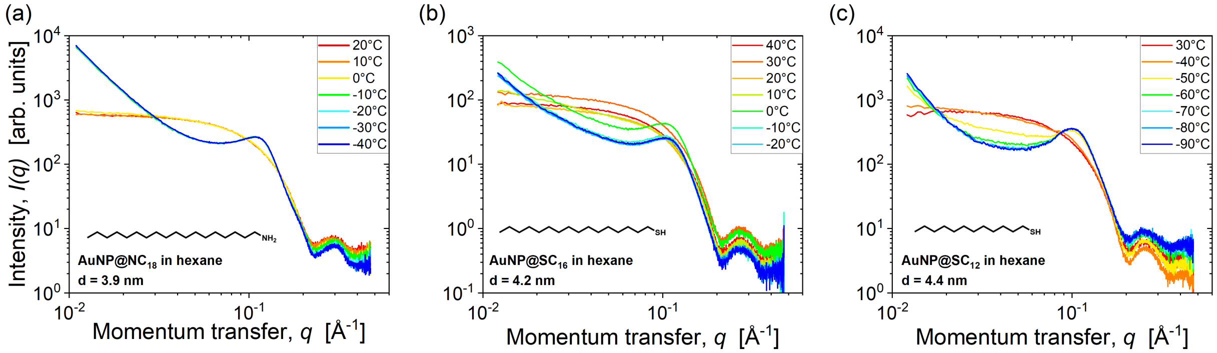

Figure S4: Temperature-dependent scattering patterns of AuNPs dispersed in hexane with a core diameter of 4.2 nm to 4.4 nm coated with linear amines and thiols: NC_{18} (a), SC_{16} (b) and SC_{12} (c).

As the scattering contrast between the organic ligands and solvent molecules is negligible, only the Au cores are visible. Therefore, the size of the cores was determined by modeling the obtained scattering patterns using the expression (see Figure S9a):

$$I(q) = P_{\mathrm{pS}}(q) + I_{\mathrm{bkg}} \tag{E1}$$

with $P_{\mathrm{pS}}(q)$ a form factor of polydisperse spheres[1] and I_{bkg} a constant accounting for background scattering. P_{pS} is given by

$$P_{\mathrm{pS}} = \int_0^\infty G(r)F(q,r)\,dr \tag{E2}$$

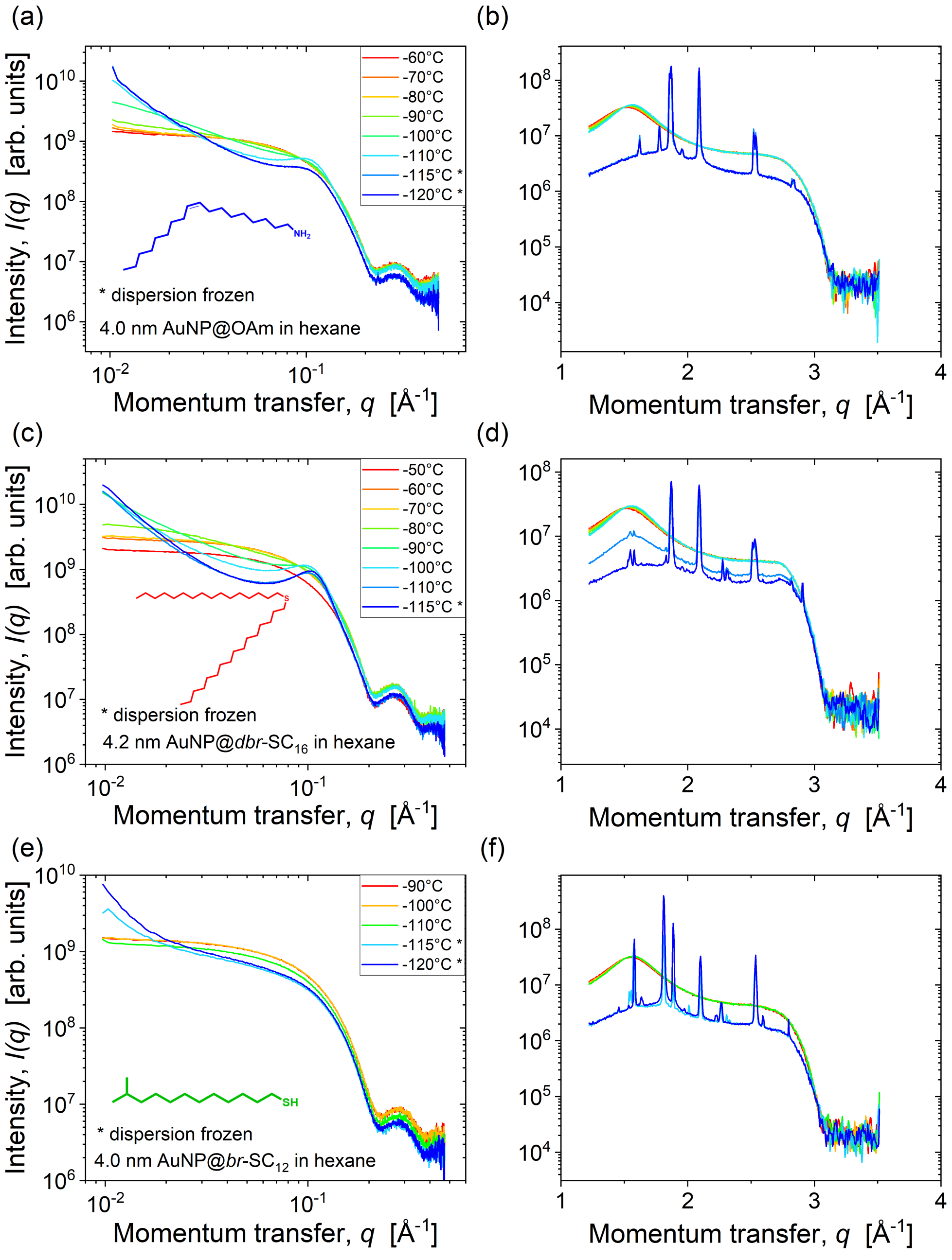

Figure S5: Temperature-dependent SAXS (a, c, e) and WAXS (b, d, f) patterns of AuNPs dispersed in hexane with a core diameter of of 4.2 nm to 4.4 nm coated with OAm ligands (a, b), *dbr*-SC_{16} ligands (c, d) branched *br*-SC_{12} ligands (e, f).

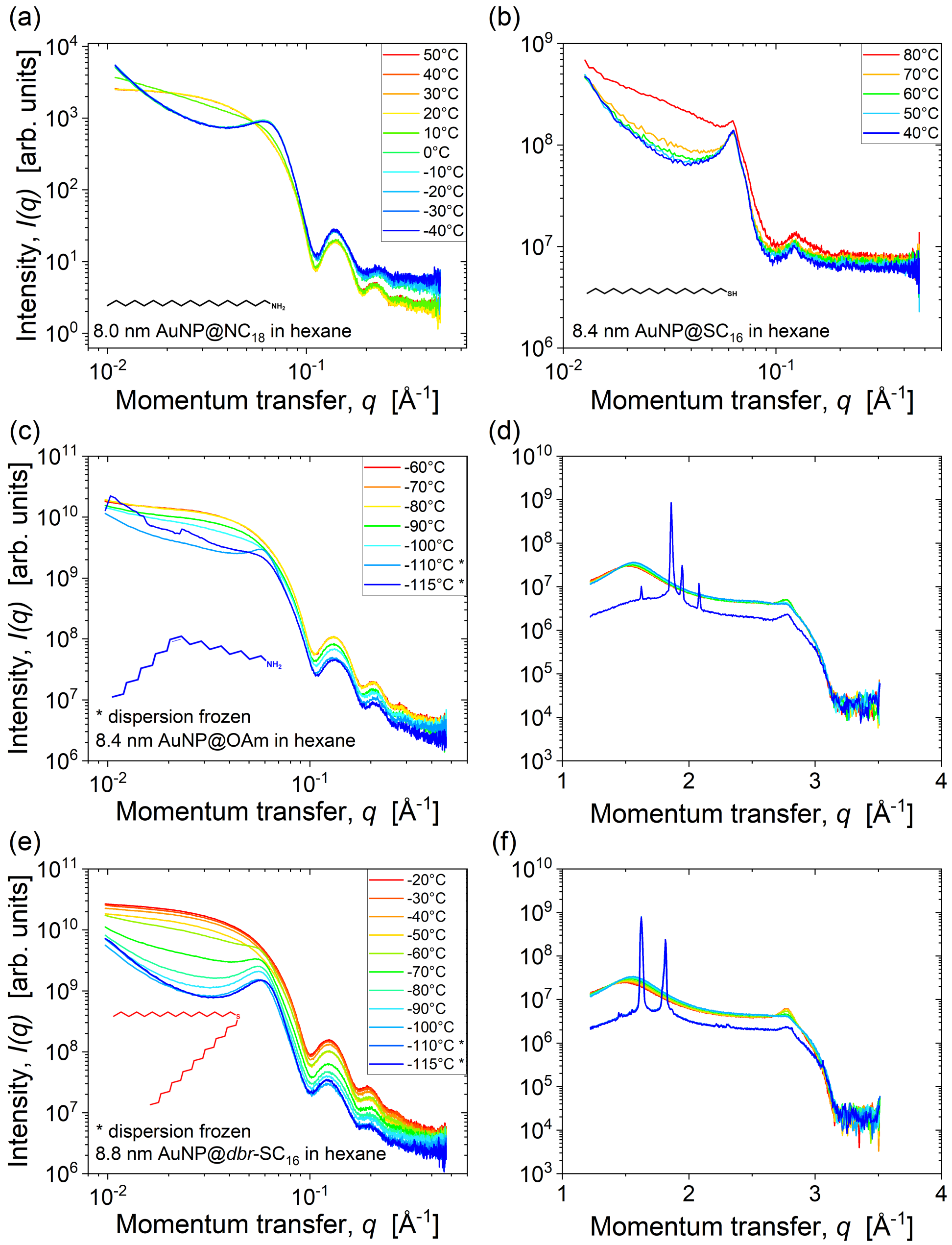

Figure S6: Temperature-dependent SAXS (a, b, c, e) and WAXS (d, f) patterns of AuNPs dispersed in hexane with a core diameter of 8.0 nm to 8.8 nm coated with NC_{18} ligands (a), SC_{16} ligands (b), OAm ligands (c, d) and *dbr*-SC_{16} ligands (e, f).

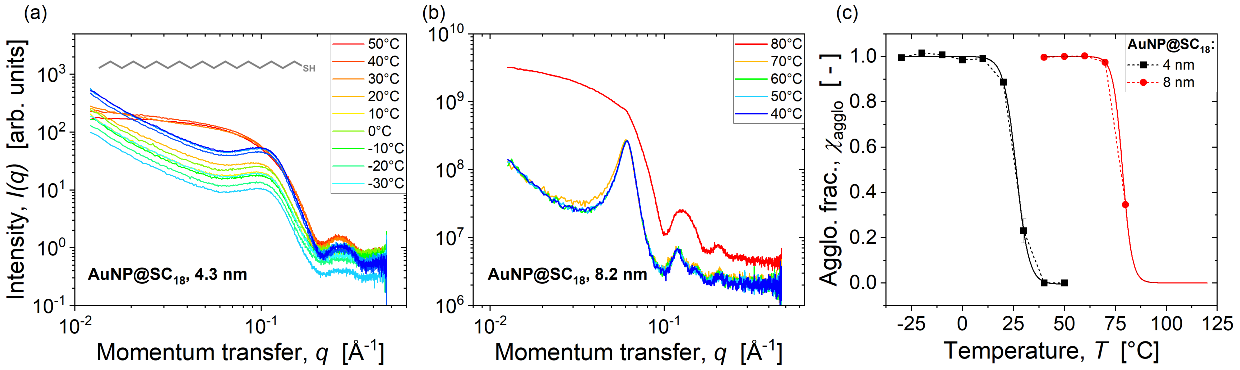

Figure S7: Temperature-dependent scattering patterns of AuNPs dispersed in hexane with core diameters of (a) 4.3 nm and (b) 8.2 nm coated with SC_{18}. (c) Temperature-dependent number fraction of AuNPs located in agglomerates, χ_{agglo}, of AuNPs coated with linear SC_{18} with core diameters of 4.3 nm (black) and 8.2 nm (red).

with r the core radius, $F(q, r)$ the form factor for monodisperse spheres and $G(r)$ the size distribution of the core radius. $F(q, r)$ is given by

$$F(q, r) = 3 \cdot \frac{\sin qr - qr \cos qr}{(qr)^3} \tag{E3}$$

$G(r_{\mathrm{c}})$ is described by a Gaussian distribution, given by

$$G(r) = \frac{1}{\sigma\sqrt{2\pi}} \exp\left[-\frac{1}{\sigma^2}(r - r_{\mathrm{avg}})^2\right] \tag{E4}$$

with r_{avg} the average core radius. σ is given by:

$$\sigma = \frac{r_{\mathrm{c}}}{r_{\mathrm{avg}}} \tag{E5}$$

The AuNPs from all batches used in this study had an r_{avg} in the range of 4.2 – 4.4 nm (σ=10 – 11 %) and 8.3 – 8.6 nm (σ=8 – 9 %). The spherical shape of the AuNPs was confirmed using transmission electron microscopy, shown in Figure S8.

The temperature-dependent SAXS patterns were analyzed following a method similar to that described by Hasan et al.[2] In this method, the SAXS patterns are modeled using the expression

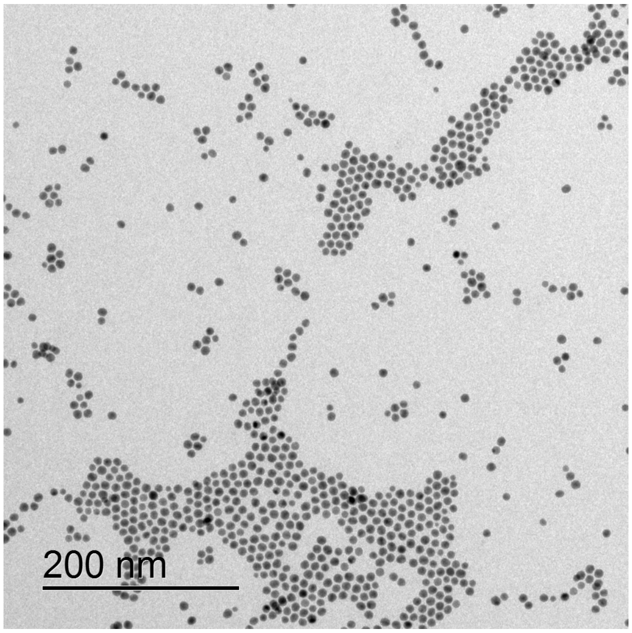

Figure S8: Transmission electron microscopy image of 8.4 nm AuNPs coated with the kinked oleylamine ligand (OAm).

$$I(q) = I_{\mathrm{P}}(q) + P_{\mathrm{pS}}(q)\left[1 - \chi_{\mathrm{agglo}} + \chi_{\mathrm{agglo}} \cdot S_{\mathrm{HS}}(q)\right] + I_{\mathrm{bkg}} \tag{E6}$$

Here, χ_{agglo} is the number fraction of AuNPs located inside agglomerates, $I_{\mathrm{P}}(q)$ is a generalized Porod law,[3] accounting for scattering by the agglomerates, and $S_{\mathrm{HS}}(q)$ is the Percus-Yevick hard-sphere structure factor[4] accounting for spatial correlation between AuNPs inside agglomerates. $S_{\mathrm{HS}}(q)$ can be calculated as[1]

$$S_{\mathrm{HS}}(q) = \frac{1}{1 + 24\eta_{\mathrm{HS}} G(2R_{\mathrm{HS}}q)/(2R_{\mathrm{HS}}q)} \tag{E7}$$

with R_{HS} the hard sphere radius and η_{HS} the hard-sphere volume fraction inside agglomerates. The function $G(x)$ is defined as:

$$\begin{aligned} G(x) = \gamma' \frac{\sin x - x\cos x}{x^2} + \delta' \frac{2x\sin x + (2 - x^2)\cos x - 2}{x^3} \\ + \epsilon' \frac{-x^4 \cos x + 4[(3x^2 - 6)\cos x + (x^3 - 6x)\sin x + 6]}{x^5} \end{aligned} \tag{E8}$$

Here, γ', δ' and ϵ' are help functions, given by

$$\gamma' = \frac{(1+2\eta)^2}{(1-\eta)^4}, \qquad \delta' = \frac{-6\eta(1+\eta/2)^2}{(1-\eta)^4}, \qquad \epsilon' = \frac{\eta\gamma'}{2} \tag{E9}$$

During fitting, r_{avg} and σ were kept fixed using the values obtained at the highest measured temperature of each respective run, i.e., where no agglomerates are present. At the lowest measured temperatures, values of χ_{agglo} of $\sim$0.8 were obtained. These were normalized to 1 to reflect the complete agglomeration expected at these temperatures.

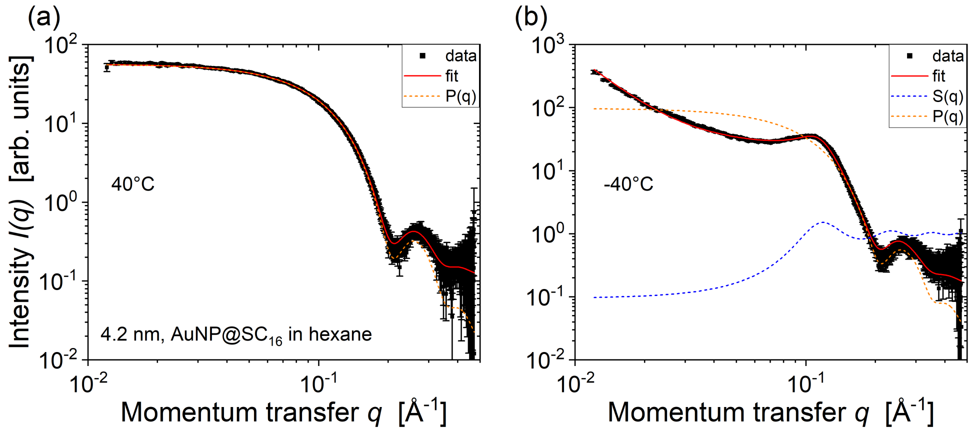

Figure S9: Scattering patterns of AuNPs with a core diameter of 4.2 nm coated with SC_{16} and dispersed in hexane at 50 °C (a) and −30 °C (b). Black dots: measured data. Red lines: fits according to eq. 1 (a) and eq. 6 (b). Dashed orange line: contribution of $P(q)$ to the model. Dashed blue line: contribution of $S(q)$ to the model.

The agglomeration temperature, T_{agglo}, defined as temperature at which χ_{agglo} equals 0.2, determined by fitting the normalized $\chi_{\text{agglo}}(T)$ using a Boltzmann sigmoid function,[5] given by

$$\chi_{\text{agglo}} = \frac{A_1 - A_2}{1 + \exp\left(\frac{T-T_0}{dT}\right)} + A_2 \tag{E10}$$

with A_1 a parameter related to plateau at high temperatures, A_2 a parameter related to plateau at low temperatures, T_0 the temperature at $\chi_{\text{agglo}} = \frac{A_1+A_2}{2}$ and dT the range in which the transition occurs.

R_{HS} can be used to calculated the core surface spacing s between gold surfaces of neigh-

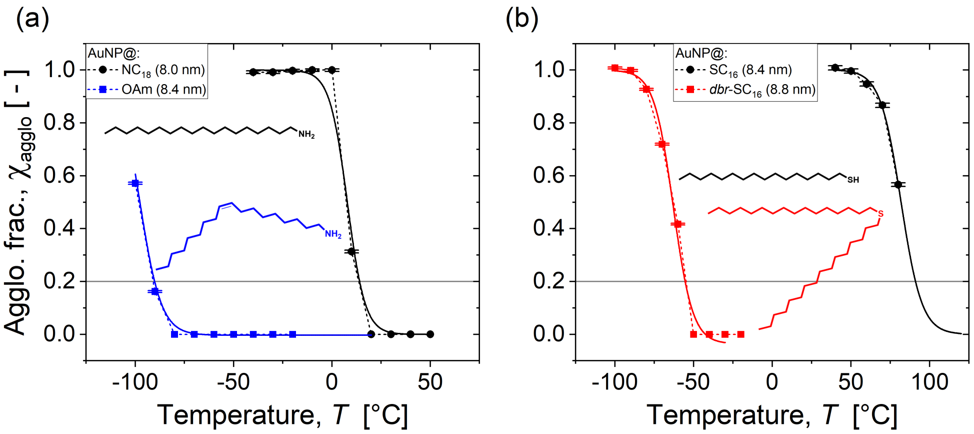

Figure S10: (a) Temperature-dependent number fraction of AuNPs located in agglomerates, χ_{agglo}, of AuNPs coated with linear NC_{18} (core diameter of 8.0 nm) and branched OAm (core diameter of 8.4 nm) and (b) linear SC_{16} (core diameter of 8.4 nm) and double-armed *dbr*-SC_{16} ligands (core diameter of 8.8 nm). The solid lines represent sigmoidal fits according to eq. 10.

boring particles in agglomerates as

$$s = 2(R_{\text{HS}} - r_{\text{avg}}) \tag{E11}$$

In previous publications, s was determined using a different method,[6,7] as is depicted in Figure S11 for 4 nm AuNPs coated with HDT ligands and dispersed in n-hexane. In a first step, $S(q)$ was determined as

$$S(q) = \frac{I(q)}{P(q)} \tag{E12}$$

with $P(q)$ the form factor of the particles determined using a measurement of dispersed AuNPs. The q value of the first intensity maximum, determined using a Lorentz distribution as depicted in Figure S11b, was related to the center-to-center distance of neighboring particles, $D_{\text{C-C}}$, following the expression

$$D_{\text{C-C}} = 1.23\frac{2\pi}{q} \tag{E13}$$

From $D_{\text{C-C}}$, the core surface spacing s was calculated as

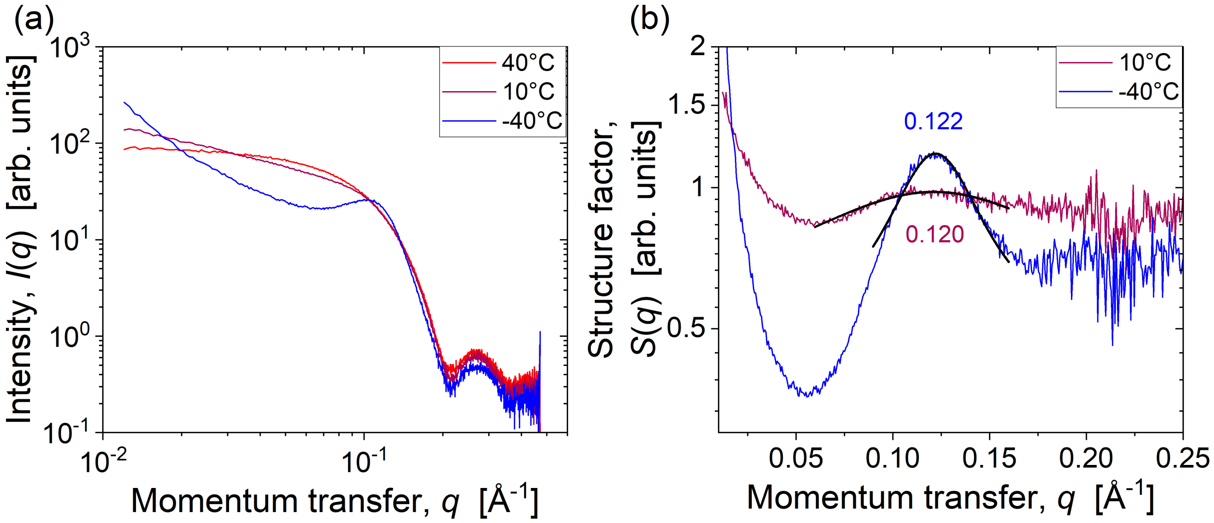

Figure S11: (a) Scattering patterns of AuNPs with a core diameter of 4 nm and coated with a HDT shell dispersed in n-hexane at 40 °C (dispersed particles), around T_{agglo} (10 °C) and at −40 °C (agglomerated particles). (b) $S(q)$ of the AuNPs around T_{agglo} and at −40 °C, calculated according to eq. 12. Solid black line: fit of the primary structure factor peak using a Lorentz distribution function.

$$s = D_{\text{C-C}} - 2r_{\text{avg}} \tag{E14}$$

In equation 13 a correction factor (1.23) was used that was established by Ehrenfest.[8] Using this method for AuNPs with a core diameter of 4 nm and coated with a HDT shell dispersed in n-hexane, a spacing (s) of 2.2 nm at T_{agglo} were determined, which is approximately 0.6 nm above that determined according to eq 6. In the work of Kister et al. a spacing 2.4 nm could be found for AuNPs with a core diameter of 4 nm coated with HDT ligands and dispersed in n-decane around T_{agglo}.[6] Consequently, the differences in between the present and previous work are due to the differences in the analysis method.

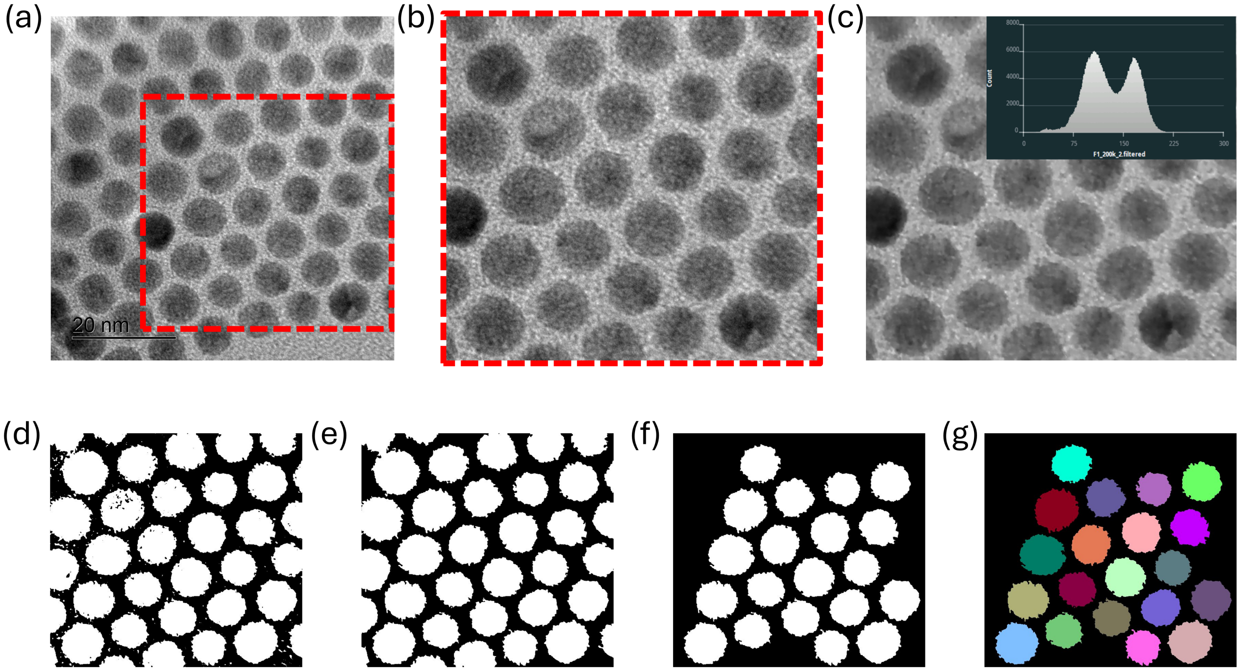

Figure S12: (a) Raw TEM image. (b) Extracted sub-image. (c) non-local means de-noised sub-image with greyscale histogram insert. (d) Segmented, binary sub-image. (e) Artifact-cleaned binary sub-image. (f) Border-killed, binary sub-image. (g) Labelled sub-image.

Analysis of the spacing of 7.3 nm AuNP@OAm using transmission electron microscopy

The TEM images were recorded at an acceleration voltage of 200 kV in bright-field imaging mode. The magnification was 200 kx and digital images (1024 × 1024 pixels) with isotropic physical pixel dimensions of 0.65 nm^2 were recorded (Figure S12a). The 8-bit greyscale images were analyzed using the software "Avizo" (Avizo 3D 2022.1, ThermoFisher Scientific, Waltham, Massachusetts, USA). Sub-images were cropped from the raw TEM images (Figure S12b) using the function "Extract Subvolume". The uneven background present in some images were corrected using the function "Background Detection Correction" which uses a B-spline to fit and correct greyscale pixel values that gradually change in the x- or y-direction of the image. To increase the signal-to-noise ratio, the greyscale images were were subsequently de-noised using a "Non-Local Means Filter" (Figure S12c). The filtered

greyscale images were segmented to obtain binary images (Figure S12d) using histogram thresholding. The thresholds were set manually at the minimum between the two peaks of the greyscale histograms (seen insert in Figure S12c). Artifacts, e.g., apparent holes within the particles or greyscale noise removed using the functions "fill holes" and "remove small spots" (Figure S12e). Particles partially located outside of the images were masked using the function "Border Kill" (Figure S12f) and the remaining particles were labeled using the function "Labeling" (Figure S12g). Pixels that belonged visibly to connected regions were assigned unique particle labels. A standard label analysis for the label images (Figure S12g) was carried out to obtain the coordinates of the particles' centers of mass. The center-to-center distances, $D_{\text{C-C}}$, between each pair of particles present were determined to obtain their pair distribution function.

This measurement on 2D particle films provides a lower bound for the core surface spacing s, because ligands in monolayers can extend above or below the plane of particle packing in a way that is not possible in 3D agglomerates.

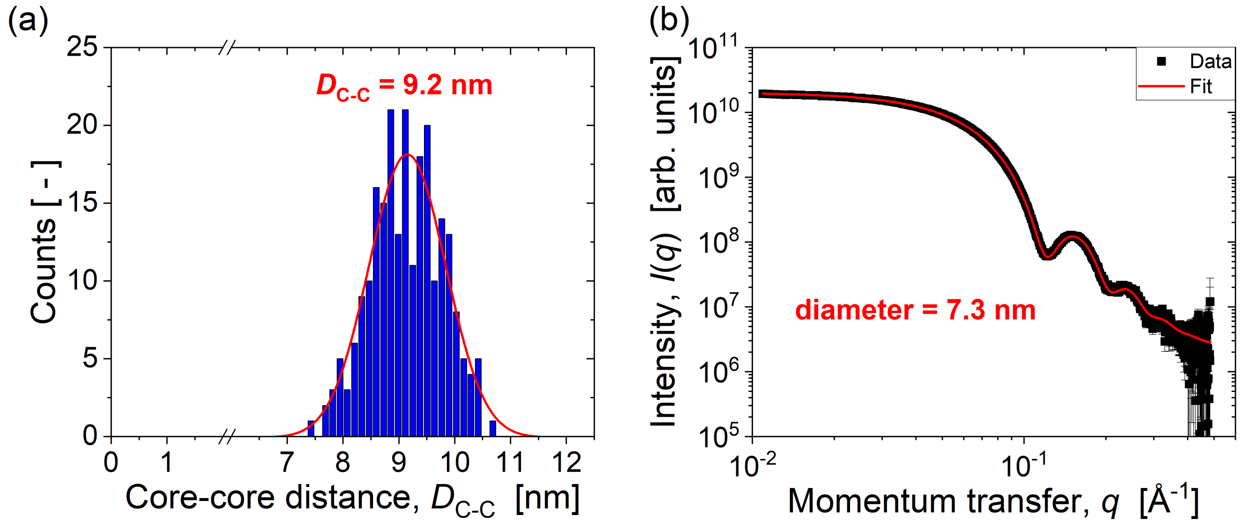

Figure S13: (a) Distribution of the center-to-center distances, $D_{\text{C-C}}$, between AuNPs with a core diameter of 7.3 nm, coated with oleylamine and arranged in a monolayer, determined using a nearest-neighbor analysis. (b) SAXS pattern of oleylamine coated AuNPs. The red line is a model fit according to eq. 1.

Four TEM images at the given microscope settings and above-described image analysis

route were analyzed, which contained a total of 105 particles. The first maximum of the combined pair distribution function, corresponding to the nearest neighbor center-to-center distances in the close-packed hexagonal arrangement of the AuNPs, is plotted in Figure S13a. A fit using a Gaussian distribution function yielded an average of $D_{\text{C-C}}$ of 9.2 nm between nearest neighbors. Using a radius of the particles of 7.3 nm, as determined using SAXS (Figure S13b), an average gold surface-to-surface distance between nearest neighbors, s, of 1.9 nm was calculated.

Thermogravimetric analysis

Thermogravimetric analysis (TGA) was used to analyze the mass of the ligand shell. The temperature-dependent changes of the mass of all particle systems are shown in Figure S14. The loss of sample mass during heating from room temperature to 1000 °C is related to the number of surface-bound ligands present, N_{ligand}, which can be determined as

$$N_{\text{ligand}} = \frac{m_{\text{loss}}}{M_{\text{ligand}}} \cdot N_{\text{A}}, \tag{E15}$$

with m_{loss} the mass loss of the sample during a TGA measurement, M_{ligand} the molar mass of the ligands, and N_{A} the Avogadro constant ($6.022 \cdot 10^{23}\text{mol}^{-1}$). Using N_{ligand}, the ligand shell density, ρ_{LS}, is calculated as

$$\rho_{\text{LS}} = \frac{N_{\text{ligand}}}{A_{\text{AuNP}}} \tag{E16}$$

where A_{AuNP} is the surface of all nanoparticles, given by

$$A_{\text{AuNP}} = A_{\text{core}} \cdot N_{\text{AuNP}}. \tag{E17}$$

where A_{core} is the surface of one nanoparticle, as calculated from the particle size, and N_{AuNP} the total number of particles present, as determined from the remaining sample mass

after the TGA measurement.

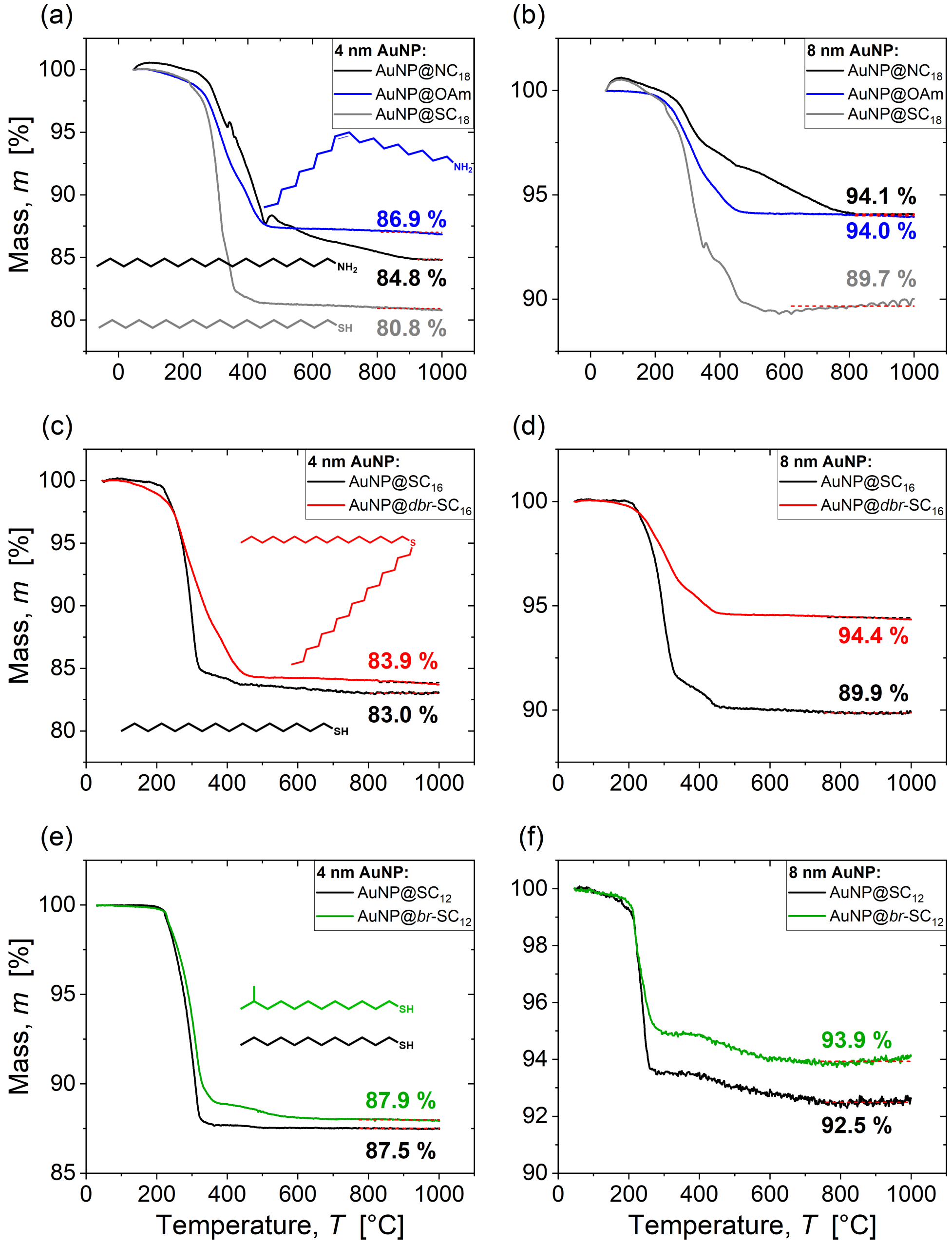

Figure S14: Thermogravimetric change of the sample mass during heating of AuNPs with a core diameter of 4 nm (a, c, e) and 8 nm (b, d, f) with linear (black curves) and non-linear ligand systems (colored curves).(a, b) AuNPs coated with NC_{18} (black) and OAm (blue) ligands. (c, d) AuNPs coated with SC_{16} (black) and *dbr*-SC_{16} (red) ligands. (e, f) AuNPs coated with SC_{12} (black) and *br*-SC_{12} (red) ligands. The dashed red or black line indicates the x-domain of each curve that was used to determine the mass loss using a linear fit function.

MD simulations

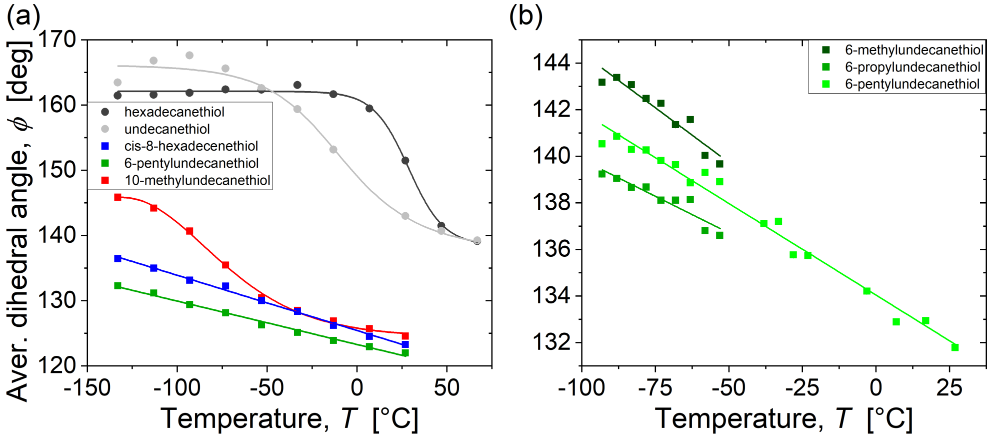

Figure S15: Average dihedral angle between CHx groups for (a) linear and branched ligands on 3.8 nm Au particles in vacuum and (b) branched ligands of varying branch lengths on 3.8 nm Au particles in hexane. Trends were fit to two-step sigmoid functions for linear and 10-methylundecanethiol ligands, and linear functions for other ligands.

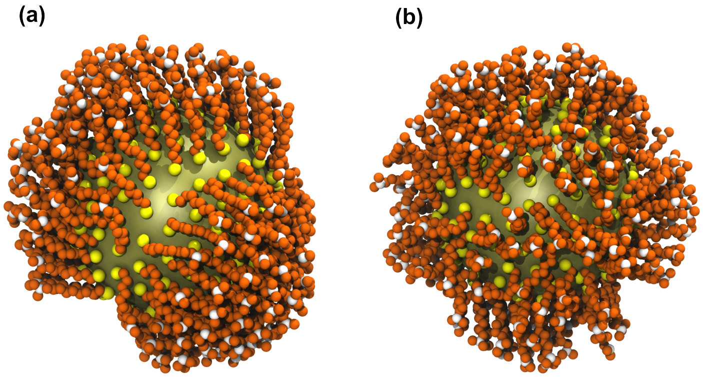

Figure S16: Simulation snapshots of a 3.8 nm Au particle coated with 10-methylundecane ligands at −93 °C in (a) vacuum and (b) hexane show ordering of ligands in vacuum.

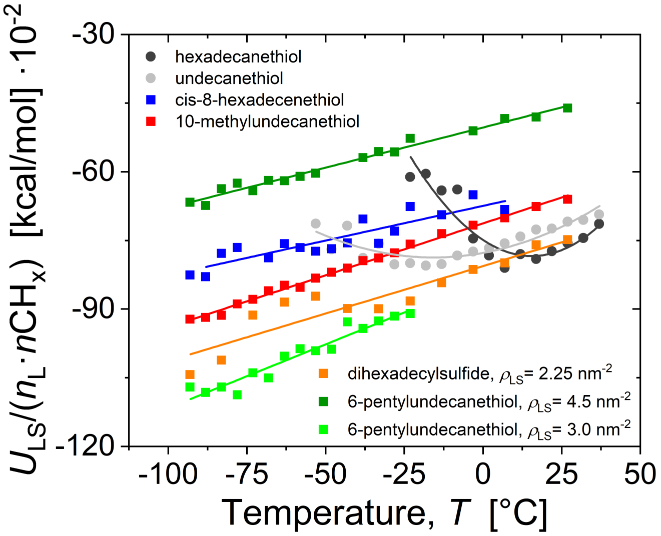

Figure S17: Ligand-solvent interaction energy for different ligands on the single 3.8 nm Au particle in hexane. The values have been normalized by the number of carbon atoms in all ligands $n_\mathrm{L}.n_\mathrm{CH}$. In order to display the trends clearly, polynomial and linear functions were fitted to the values.

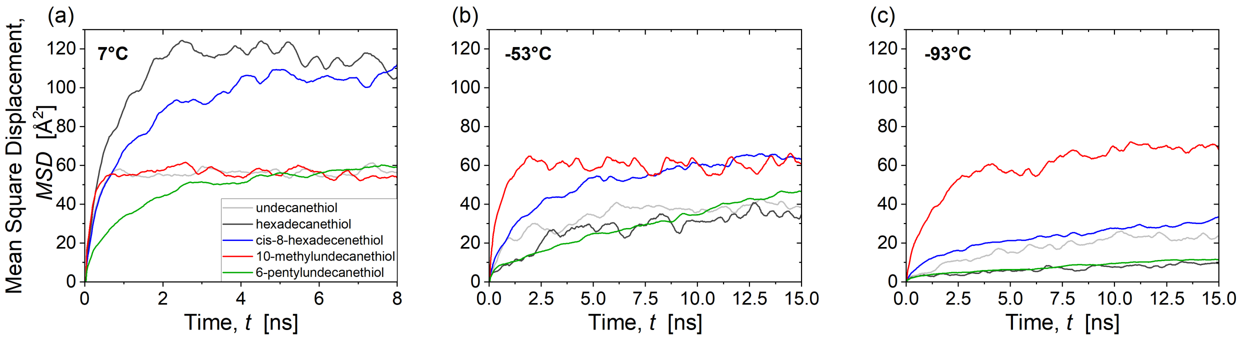

Figure S18: Comparison of ligand mobilities at (a) 7 °C, (b) −53 °C, and (c) −93 °C. The plots show the mean square displacement of ligand atoms as a function of time. To eliminate noise and enhance visualization, the curves were smoothed using discrete convolution.

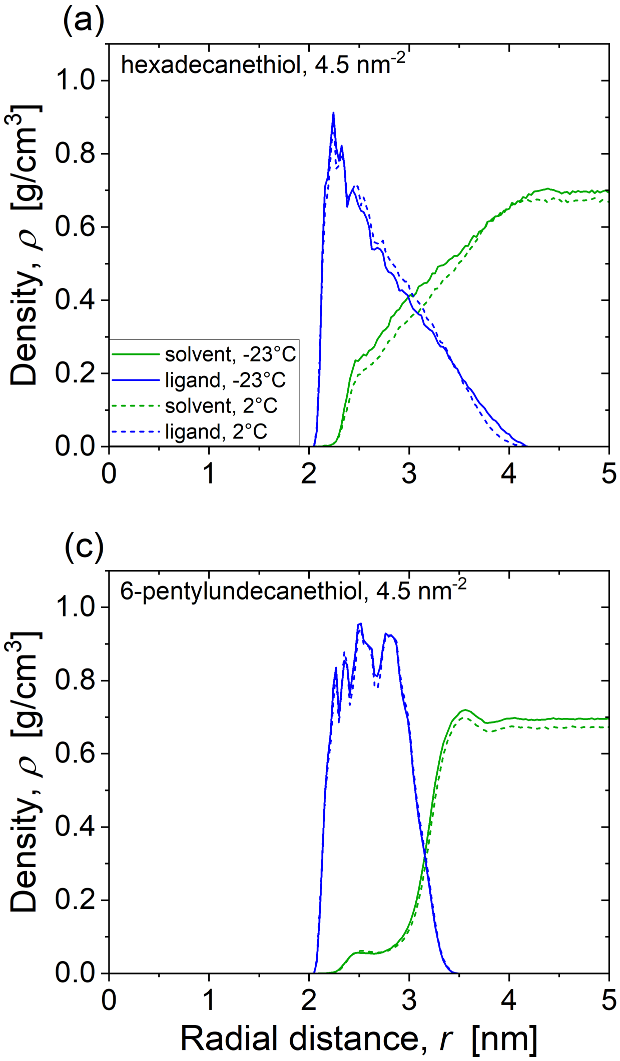

Figure S19: Radial density distribution as a function of the distance r from the centre of the Au core for ligand and solvent molecules at two different temperatures for (a) hexadecanethiol, (b) cis-8-hexadecenethiol and (c) 6-pentylundecanethiol, with 4.5 nm^{-2} density and (d) 6-pentylundecanethiol with 3 nm^{-2} density.

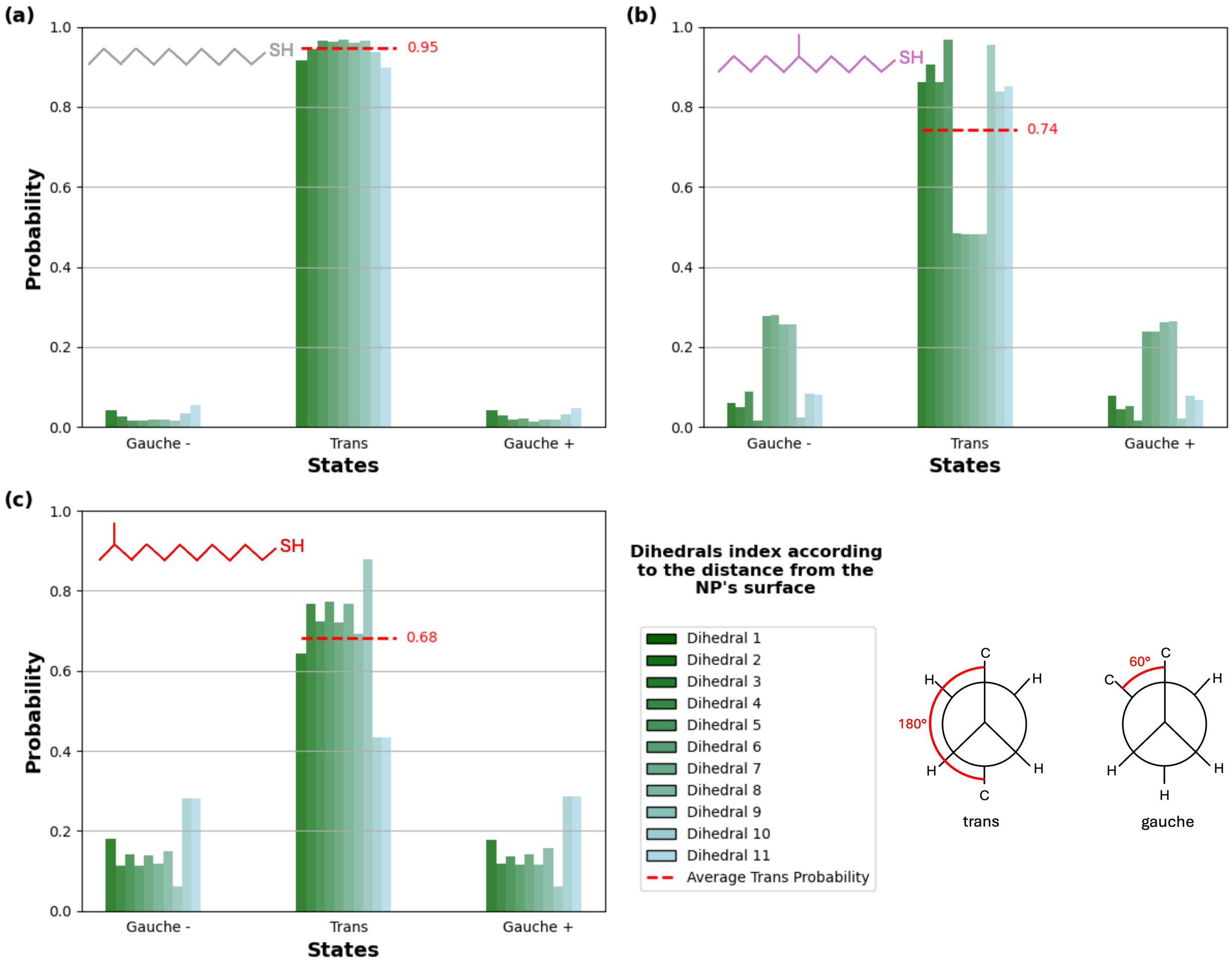

Figure S20: Probability distribution functions of dihedral states observed for ligand shells composed of (a) undecane, (b) 6-methylundecane, and (c) 10-methylundecane. The dihedrals are indexed based on their distance from the nanoparticle surface, with Dihedral 1 corresponding to the S–C–C–C dihedral in case. The gauche+, trans and gauche- states consist of all angles from 0°–120°, 120°–240°, and 240°–360°, respectively. The red dashed lines indicate the average probability of observing a dihedral angle belonging to the trans state.

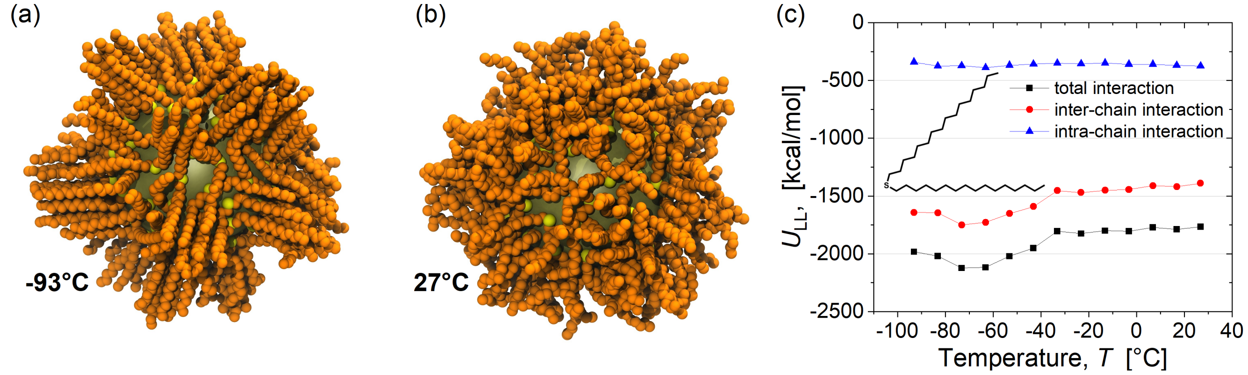

Figure S21: Simulation snapshots of a 3.8 nm Au particle coated with dihexadecylsulfide ligands at (a) −93 °C and (b) 27 °C, showing ordering of ligands into small bundles at low temperature. (c) The ligand-ligand interaction energy as a function of temperature, decomposed into intra and inter ligand contributions, shows that bundle formation coincides with an increase only in the inter ligand interaction energy. This is consistent with the snapshots showing that the bundles are composed of only one tail from each ligand, which limits their size.

References

(1) Pusey, P. In *Neutrons, X-rays and Light: Scattering Methods Applied to Soft Condensed Matter*; Lindner, P., Zemb, T., Eds.; North-Holland, 2002; Chapter Introduction to Scattering Experiments.

(2) Hasan, M. R.; Niebuur, B.-J.; Siebrecht, M.; Kuttich, B.; Schweins, R.; Widmer-Cooper, A.; Kraus, T. The Colloidal Stability of Apolar Nanoparticles in Solvent Mixtures. *ACS Nano* **2023**, *17*, 9302–9312.

(3) Porod, G. Die Röntgenkleinwinkelstreuung von dichtgepackten kolloiden Systemen. *Kolloid-Zeitschrift* **1951**, *124*, 83–114.

(4) Percus, J. K.; Yevick, G. J. Analysis of Classical Statistical Mechanics by Means of Collective Coordinates. *Phys. Rev.* **1958**, *110*, 1–13.

(5) Knapp, T. V.; Hasan, M. R.; Niebuur, B.-J.; Widmer-Cooper, A.; Kraus, T. Stabilization of Apolar Nanoparticle Dispersions by Molecular Additives. *Langmuir* **2024**, *40*, 13527–13537.

(6) Kister, T.; Monego, D.; Mulvaney, P.; Widmer-Cooper, A.; Kraus, T. Colloidal Stability of Apolar Nanoparticles: The Role of Particle Size and Ligand Shell Structure. *ACS Nano* **2018**, *12*, 5969–5977.

(7) Monego, D.; Kister, T.; Kirkwood, N.; Doblas, D.; Mulvaney, P.; Kraus, T.; Widmer-Cooper, A. When Like Destabilizes Like: Inverted Solvent Effects in Apolar Nanoparticle Dispersions. *ACS Nano* **2020**, *14*, 5278–5287.

(8) Guinier, A. *X-ray Diffraction in Crystals, Imperfect Crystals, and Amorphous Bodies*; Dover Books on Physics Series; Dover Publications, 1994.
